# Characterization of Chromatin Accessibility and Gene Expression upon Cold Stress Reveals that the RAV1 Transcription Factor Functions in Cold Response in *Vitis Amurensis*

**DOI:** 10.1093/pcp/pcab115

**Published:** 2021-07-19

**Authors:** Chong Ren, Huayang Li, Zemin Wang, Zhanwu Dai, Fatma Lecourieux, Yangfu Kuang, Haiping Xin, Shaohua Li, Zhenchang Liang

**Affiliations:** Beijing Key Laboratory of Grape Sciences and Enology, Key Laboratory of Plant Resource, Institute of Botany, Chinese Academy of Sciences, 20 Nanxincun, Xiangshan, Beijing 100093, PR China; Beijing Key Laboratory of Grape Sciences and Enology, Key Laboratory of Plant Resource, Institute of Botany, Chinese Academy of Sciences, 20 Nanxincun, Xiangshan, Beijing 100093, PR China; University of Chinese Academy of Sciences, 19 Yuquan Rd, Beijing 100049, PR China; Beijing Key Laboratory of Grape Sciences and Enology, Key Laboratory of Plant Resource, Institute of Botany, Chinese Academy of Sciences, 20 Nanxincun, Xiangshan, Beijing 100093, PR China; Beijing Key Laboratory of Grape Sciences and Enology, Key Laboratory of Plant Resource, Institute of Botany, Chinese Academy of Sciences, 20 Nanxincun, Xiangshan, Beijing 100093, PR China; EGFV, Bordeaux Sciences Agro, INRA, Université de Bordeaux, ISVV, 210 chemin de Leysotte, Villenave d’Ornon 33882, France; Beijing Key Laboratory of Grape Sciences and Enology, Key Laboratory of Plant Resource, Institute of Botany, Chinese Academy of Sciences, 20 Nanxincun, Xiangshan, Beijing 100093, PR China; University of Chinese Academy of Sciences, 19 Yuquan Rd, Beijing 100049, PR China; Key Laboratory of Plant Germplasm Enhancement and Specialty Agriculture, Wuhan Botanical Garden, Chinese Academy of Sciences, 1 Lumo Rd, Wuhan 430074, PR China; Beijing Key Laboratory of Grape Sciences and Enology, Key Laboratory of Plant Resource, Institute of Botany, Chinese Academy of Sciences, 20 Nanxincun, Xiangshan, Beijing 100093, PR China; Beijing Key Laboratory of Grape Sciences and Enology, Key Laboratory of Plant Resource, Institute of Botany, Chinese Academy of Sciences, 20 Nanxincun, Xiangshan, Beijing 100093, PR China

**Keywords:** ATAC-seq, Cold response, RAV1, RNA-seq, Transcription factor, *Vitis amurensis*

## Abstract

Cold tolerance is regulated by a variety of transcription factors (TFs) and their target genes. Except for the well-characterized C-repeat binding factors (CBFs)-dependent transcriptional cascade, the mechanisms of cold tolerance mediated by other transcriptional regulatory networks are still largely unknown. Here, we used the assay for transposase-accessible chromatin with sequencing (ATAC-seq) and RNA-seq to identify cold responsive TFs in *Vitis amurensis*, a grape species with high cold hardiness. Nine TFs, including CBF4, RAV1 and ERF104, were identified after cold treatment. Weighted gene co-expression network analysis (WGCNA) and gene ontology (GO) analysis revealed that these TFs may regulate cold response through different pathways. As a prime candidate TF, overexpression of *VaRAV1* in grape cells improved its cold tolerance. The transgenic cells exhibited low electrolyte leakage and malondialdehyde content and high peroxidase activity. Moreover, the TF gene *TCP8* and a gene involving in homogalacturonan biosynthesis were found to be regulated by VaRAV1, suggesting that the contribution of VaRAV1 to cold tolerance may be achieved by enhancing the stability of cell membrane and regulating the expression of target genes involved in plant cell wall composition. Our work provides novel insights into plant response to cold stress and demonstrates the utility of ATAC-seq and RNA-seq for the rapid identification of TFs in response to cold stress in grapevine. VaRAV1 may play an important role in adaption to cold stress.

## Introduction

Environmental conditions have a significant effect on plant growth, development and ecological distribution. As one of the most severe environmental factors, low temperature adversely attenuates the normal functions of plant physiological processes and, sometimes, may cause permanent injuries or death (Thomashow 1999, Puhakainen et al. 2004, Wisniewski et al. 2004). Most temperate plants can acquire freezing tolerance by a process called cold acclimation, during which plants are exposed to low but nonfreezing temperature (Chinnusamy et al. 2003). Cold acclimation involves multiple physiological and biochemical pathways (Thomashow 1999), and the tolerance to cold stress, including chilling (<10°C) and freezing (<0°C) stress, is regulated by a variety of genes (Kreps et al. 2002). In *Arabidopsis*, the cold-regulated genes have been estimated to account for 4% to 20% of the genome (Lee et al. 2005, Hannah et al. 2005).

The mechanism of cold tolerance in plants has been widely studied in the past two decades. A considerable number of *cold-responsive* (*COR*) genes and their corresponding regulatory networks have been identified in different plant species, especially in the model plant *Arabidopsis thaliana* (Lee et al. 2005). The C-repeat (CRT) binding factors (CBFs), which are also known as dehydration-responsive element binding factor 1 transcription factors (DREB1s), have been revealed to play crucial roles in cold response (Stockinger et al. 1997, Liu et al. 1998). CBF proteins induce the expression of *COR* genes by binding to the CRT elements on the promoters of *COR* genes under cold stress (Gilmour et al. 1998, Jia et al. 2016, Zhao et al. 2016). The *COR* genes involve processes in membrane transport, transcription, hormone metabolism and signaling, osmolyte biosynthesis, reactive oxygen species (ROS) detoxification and many other cellular protective processes (Chinnusamy et al. 2007), and their expression results in enhanced plant freezing tolerance. The expression of *CBFs* is regulated by several transcription factors (TFs). The Inducer of CBF expression 1 (ICE1) is considered as a chief regulator of *CBFs* in *Arabidopsis* (Chinnusamy et al. 2003). This MYC-type basic helix-loop-helix TF can bind to canonical MYC *cis*-elements on the promoters of *CBFs* and activate their expression (Chinnusamy et al. 2003, Ding et al. 2015). ICE1 is posttranslationally regulated by high expression of osmotically responsive gene 1 (HOS1), open stomata 1 (OST1) and SAP/MIZ (SIZ1; Dong et al. 2006, Miura et al. 2007, Ding et al. 2015). Recently, mitogen-activated protein kinase 3 (MPK3) and MPK6 were found to destabilize ICE1 through phosphorylation, which reduces the transcriptional activity of ICE1 (Li et al. 2017, Zhao et al. 2017). Additionally, previous reports also underlined the induction by cold stress of an abscisic acid (ABA)-dependent signal transduction cascade. For instance, exogenous ABA treatment could enhance a number of genes that respond to cold stress (Shinozaki et al. 2003).

As one of the most widely cultivated fruit crops, grape (*Vitis* L.) holds a worldwide economic importance. However, cold stress greatly restricts the geographic distribution of grapevine cultivars, hinders its growth and development and seriously decreases berry production and quality. Significantly, the currently dominate cultivars used for the production of wine are derived from *V. vinifera*, which is sensitive to low temperature in winter (Zhang and Dami 2012). In contrast, amur grape (*Vitisamurensis*), a Chinese wild-growing grape, is extremely cold tolerant. The prominent cold hardiness of *V. amurensis* makes it a valuable germplasm resource for grape cold-tolerant breeding. According to previous transcriptome studies, we found that ethylene and raffinose partially contribute to cold tolerance of *V. amurensis* by modulating the expression of *Ethylene Response Factor 057* (*VaERF057*), *VaERF092* and *AQUILO* (AcQUIred tolerance to LOw temperatures) genes, respectively (Sun et al. 2016, 2018, 2019). However, the molecular basis of the remarkable tolerance of *V. amurensis* to cold stress remains largely unknown.

Recently, a simple but sensitive assay for transposase-accessible chromatin with sequencing (ATAC-seq) was described (Buenrostro et al. 2013). ATAC-seq employs a hyperactive Tn5 transposase that cleaves genomic DNA and adds sequencing adapters simultaneously. The digested fragments deriving from open chromatin can be converted into a high-throughput sequencing library by polymerase chain reaction (PCR). Mapping the readout of sequencing library allows for identifying highly accessible chromatin regions and putative TF-binding sites (Lu et al. 2017). Due to the simple procedure and low material input, ATAC-seq has been swiftly applied in *Arabidopsis*, rice, tomato, *Medicago truncatula* and wheat to assay plant DNA regulatory regions (Wilkins et al. 2016, Bajic et al. 2017, Lu et al. 2017, Sijacic et al. 2018, Maher et al. 2018, Frerichs et al. 2019, Concia et al. 2020). In this study, we employed ATAC-seq approach to identify accessible chromatin regions in *V. amurensis* at an early stage (2 h) of cold treatment (4°C). According to the ATAC-seq results, we identified 1,565 transposase hypersensitive sites (THSs) that were differentially enriched after cold treatment. Motif analysis with these THSs combined with the RNA-seq results highlighted nine cold-responsive TFs, including the well-known TF CBF4. The TF-gene co-expression networks were developed using weighted gene co-correlation network analysis (WGCNA). Based on the results of WGCNA and gene ontology (GO) analysis, we found that ethylene signaling pathway mediated by the identified ERF TFs, such as RAV1, ERF104 and ERF1A, plays a significant role in cold response in *V. amurensis*. Furthermore, the overexpression of *VaRAV1* in grape cells improved its cold tolerance. Transient luciferase (Luc) activity and yeast one-hybrid (Y1H) assays revealed that VaRAV1 may enhance cold tolerance by positively regulating the expression of other TFs and the gene involved in plant cell wall composition. Our results provide novel insights into the signaling during cold response and demonstrate that ATAC-seq combined with RNA-seq could be a powerful tool for the quick identification of novel cold-responsive TFs in grapevine.

## Results

### Cold-responsive pathways had been triggered at 2 h of cold treatment

**Fig. 1:**
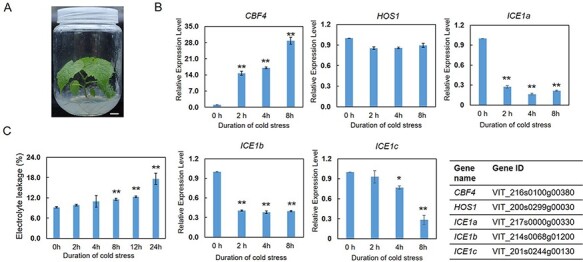
Characterization of gene expression and electrolyte leakage in *in vitro Vitis amurensis* plants during cold treatment. (A) *In vitro V. amurensis* plant used for cold treatment. One-month-old plants with uniform growth status were used for cold treatment. Scale bar corresponds to 1 cm. (B) The expression profiles of cold-responsive genes during cold treatment. The relative expression levels of *CBF4, HOS1, ICE1a, ICE1b* and *ICE1c* were investigated at 0, 2, 4 and 8 h of cold treatment, respectively, using quantitative real-time PCR (qPCR). The gene IDs are listed in the table. (C) Electrolyte leakage analysis of *V. amurensis* leaves after cold treatment. Values are means of three replicates ± SD. The test of significance was performed using Student’s *t*-test. *P < 0.05; **P < 0.01.

One-month-old *in vitro* subcultured plants of *V. amurensis* with uniform growth status were used for cold treatment (**Fig. 1A**). To make sure that the cold-responsive pathways had been triggered, we first characterized the expression profiles of some well-known marker genes during the cold treatment. The results of quantitative real-time PCR (qPCR) revealed a rapid and strong expression induction of *CBF4* at 2 h upon cold stress (**Fig. 1B**), whereas the expressions of *CBF1, 2, 3* were not detected due to the lack of valid values (data not shown). The possible reason is that the expressions of grape *CBF1, 2, 3*, as well as their expression patterns in different tissues and cultivars, were more variable during cold stress, whereas the expression of *CBF4* was relatively stable (Xiao et al. 2006, 2008). Moreover, different CBF members may have different functions, given the fact that drought tolerance of *Arabidopsis* was most increased by the grape *CBF1* (Siddiqua and Nassuth 2011). The transcript level of *ICE1a* and *ICE1b* was decreased, while the transcript abundance of *HOS1* and *ICE1c* was still constant at 2 h after exposure to cold stress (**Fig. 1B**). According to the previous results, ICE1 and HOS1 were mainly posttranslationally regulated, and the normal function of these TFs highly depends on protein stability (Dong et al. 2006, Miura et al. 2007, Ding et al. 2015, Li et al. 2017, Zhao et al. 2017). Furthermore, the analysis of electrolyte leakage, which is generally used as an indicator of cell membrane injuries, revealed that cold-induced electrolyte leakage obviously increased from 8 h of cold stress in grape leaves (**Fig. 1C**). Altogether, these results indicated that transcriptional networks involving the typical ICE1-CBF transcriptional cascade had been triggered at 2 h of cold treatment when plant cell membranes were still undamaged. Hence, we decided to use the samples collected at 2 h of cold treatment for subsequent ATAC-seq and RNA-seq analyses.

### Landscape of accessible chromatin regions in grapevine

Six libraries prepared from three biological replicates under control (26°C) and cold treatment (4°C), respectively, were obtained through paired-end sequencing (**Supplementary Table S2**). Most of the ATAC-seq reads were reproducible among three biological replicates (**Supplementary Fig. S1**). Over 67% of total reads could be uniquely mapped to the genome by aligning reads to the grape 12X genome (version 2.1; **Supplementary Table S2**). For each library, the fragment length distribution of aligned reads was analyzed to check the number of nucleosome-free reads (<150 bp) and nucleosome-containing reads (>150 bp; Sijacic et al. 2018). The results showed that the fragment length distribution of reads was primarily around 100 bp or smaller (**Supplementary Fig. S2**), indicating that the prepared ATAC-seq libraries were mainly composed of nucleosome-free reads. Nucleosome-free reads are accessible chromatin regions where TFs might bind, whereas nucleosome-containing reads were relatively less accessible to TF binding (Sijacic et al. 2018). In short, our ATAC-seq datasets could be used for the identification of accessible chromatin regions in *V. amurensis*.

**Fig. 2:**
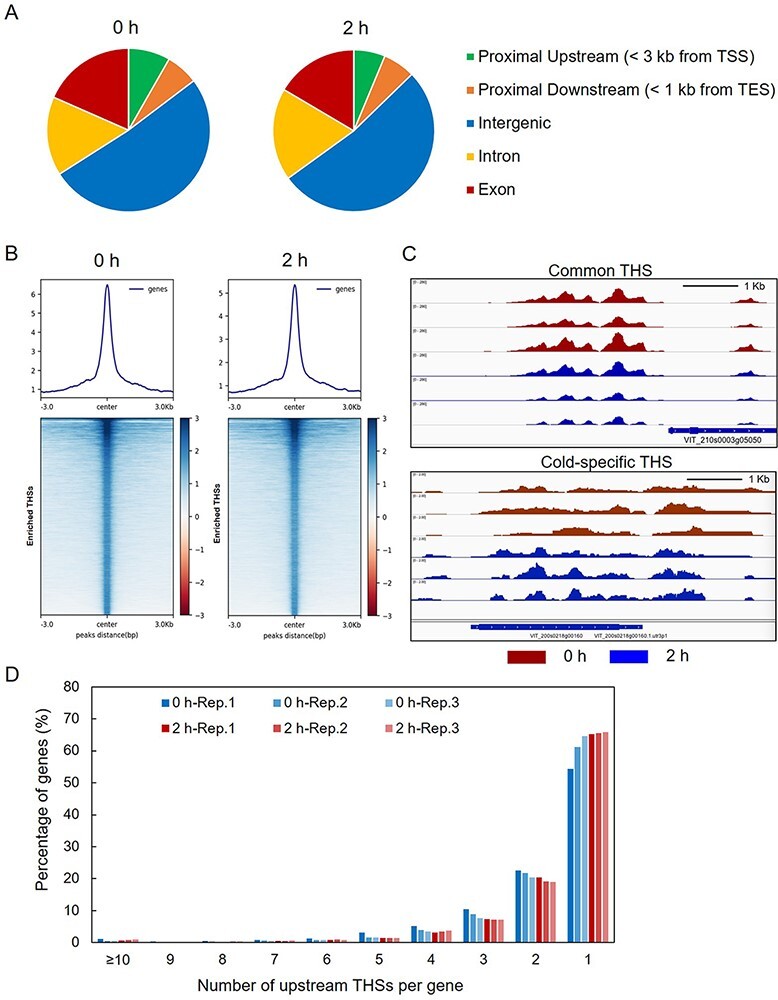
Overview of assay for transposase-accessible chromatin (ATAC-seq) data in *V. amurensis*. (A) Genomic distributions of all the THSs identified in ATAC-seq data. (B) Average plots and heatmaps of ATAC-seq signals of over-enriched THSs at 0 h (left panel) and 2 h (right panel) of cold treatment. (C) Examples of Integrated Genome Viewer (IGV) snapshots of normalized ATAC-seq reads from samples collected at 0 h (red) and 2 h (blue). The common transposase hypersensitive sites (THSs) identified at both 0 h and 2 h (upper) and cold-specific THSs differentially enriched at 2 h (lower) were observed. (D) Number of upstream THSs per gene in *V. amurensis*. Graph exhibits the percentage of genes with a given number of upstream THSs.

The called peaks, or THSs, denote the enriched accessible chromatin regions. Contrary to *Arabidopsis*, in which the majority of THSs were located in the upstream of transcription start site (TSS; Maher et al. 2018, Sijacic et al. 2018), most of THSs in *V. amurensis* were found to be located within gene body (exon and intron, 34%) or located in the intergenic regions (52%). Around 8% of THSs were located within 3 kb upstream of TSS, and only 6% were located within 1 kb downstream of gene transcription end site (TES; **Fig. 2A**). This distribution pattern is similar to that in tomato (*Solanum lycopersicum*; Maher et al. 2018). The possible reason of this distribution pattern in grapevine is due to the big genome size. The proportion of THSs between proximal upstream and intergenic regions varied remarkably with genome sizes or, rather, the amount of intergenic space in the species genome (Maher et al. 2018). We next examined the signal of those THSs located within 3 kb of TSS using heatmaps and average plots. The results revealed that the enriched THSs at both 0 h and 2 h exhibited the strongest signal around the TSS center (**Fig. 2B**), suggesting that the regions near TSS are accessible. Through ATAC-seq assay, we identified both cold-specific THSs that differentially enriched at 2 h after cold treatment and common THSs, which were detected under both control and cold conditions (**Fig. 2C**).

The number of THSs that were used to identify one gene in grapevine was also characterized. For instance, we obtained 18,770 THSs from the replicate 1 library of 0 h (0 h-Rep.1) by peak calling (**Fig. 2D**), and a total of 9,042 genes were obtained after mapping these THSs to grape genome. The number of THSs per gene can be calculated, and the majority of mapped genes were only associated with one upstream THS (**Fig. 2D**), which is consistent with the previous finding in *Arabidopsis* (Maher et al. 2018).

### Enriched motif analysis and identification of TFs in response to cold stress

**Fig. 3:**
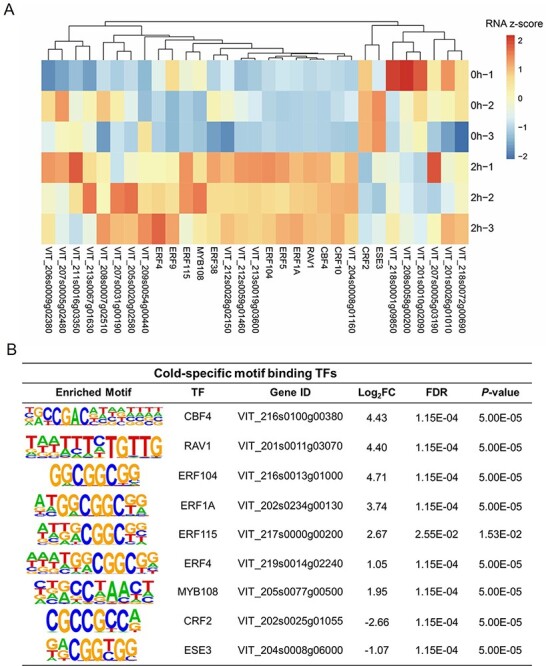
The candidate transcription factors (TFs) in response to cold stress. (A) A heatmap of expression profiles of TF genes after cold treatment. The TFs identified from motif analysis based on the differentially enriched transposase hypersensitive sites after cold treatment were further evaluated according to their expression upon cold treatment. The expression data of TFs genes obtained from RNA-seq were normalized using Z-score. (B) Nine TFs that most likely function in early cold response. The identified motifs, the corresponding TFs and their best matches in grape, log2 fold changes (log2FC) of gene expression, FDR and *P* values are shown. Only those TFs genes with a log2FC >1 or <−1 and an FDR value <0.05 were retained.

The identified THSs can be used to predict motifs generally recognized by TFs, and the position of THSs or predicted motifs indicates the possible binding sites of TFs. Since quantitative differences are prone to being omitted from analysis using all-or-nothing peak-calling approach (Sijacic et al. 2018), we, therefore, screened the differentially enriched THSs after cold treatment based on the evaluation of read counts of each THS using DEseq2 (Love et al. 2014). As a result, we identified 1,376 positively enriched and 189 negatively enriched THSs at 2 h after cold treatment (**Supplementary Fig. S3**). These THSs were adopted for motif analysis by using the ‘Find Motifs’ function of HOMER, and 77 unique motifs were thereby identified (**Supplementary Figs. S4–S5**). By searching the plant TFs (motifs) database, we could know the TFs that probably recognize the specific motifs. As expected, the motifs for the key TFs CBF1, 2, 3 and 4 were identified within the 77 motifs, suggesting that CBFs may play important roles in cold response in grape (**Supplementary Fig. S4**). Furthermore, a number of TFs belonging to IDD, GATA factor, zinc finger TF family, MYB TF family and AP2/ERF TF superfamily were also identified as candidates controlling intracellular responses to cold stress (**Supplementary Fig. S4**). Given that the public data of plant TF-binding profiles are mainly collected from the model plant species *Arabidopsis*, we thus identified grape TFs using BLAST search by taking advantage of conservative property of predicted motifs and homologous TFs proteins between different species. This analysis led to the selection of 51 grape TFs after removing the redundant and unannotated TFs (Supplementary Dataset 1). Combining the RNA-seq results, we finally obtained 31 TFs genes with altered expression after cold treatment (**Fig. 3A**). Although most of these genes could be induced or repressed by cold, only nine TFs genes, including the cold-responsive gene *CBF4*, exhibited significant changes [an absolute value of log2 fold change (log2FC) >1 and a *P* value <0.05] in their expression (**Fig. 3B**). All the nine TFs genes were up-regulated by cold treatment except for *CRF2* and *ESE3*. The expression of the two genes was obviously decreased by cold stress (**Fig. 3B**). In contrast, *CBF4, RAV1* and the *ERF* gene *ERF104* were strongly induced (log2FC >4) by cold (**Fig. 3B**).

### WGCNA and GO analysis of the genes associated with cold-responsive TFs

**Fig. 4:**
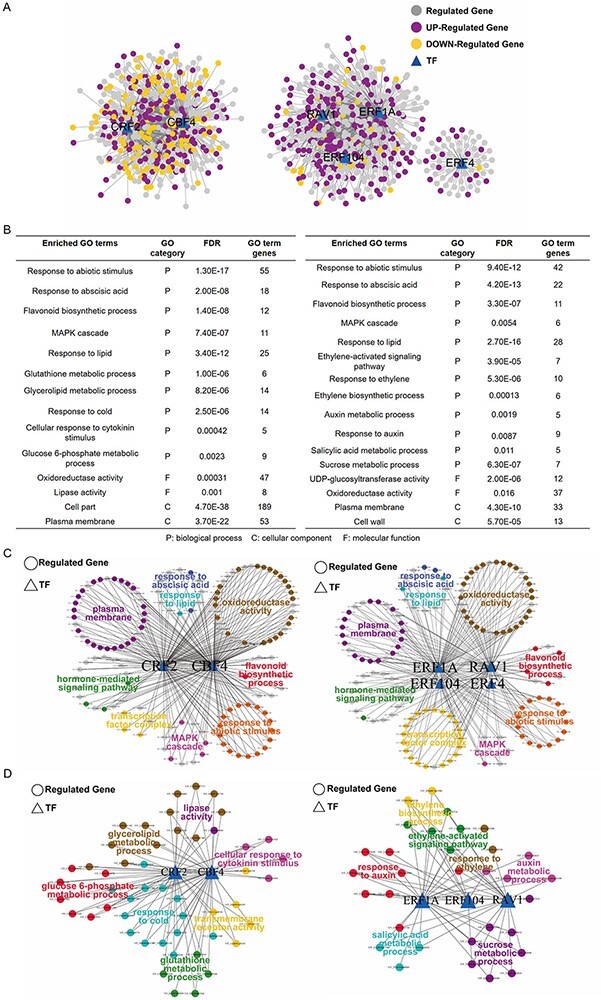
Gene ontology (GO) analysis of the enriched transcription factors (TFs) and their target genes. (A) Gene co-expression networks. The six out of the nine TFs shown in **Fig. 3B** were divided into two independent regulatory networks (CBF4-CRF2 and RAV1-ERFs) using the weighted gene co-expression network analysis (WGCNA). The TFs are indicated by triangles while the target genes are denoted by circles. The differentially expressed genes (DEGs) are indicated in purple (up-regulated) and orange (down-regulated), respectively. (B) Enriched GO terms for the TFs and their target DEGs shown above in (A). Enriched GO terms for the CBF4-CRF2 and RAV1-ERFs networks are shown on the left and right, respectively. (C) The common GO terms shared by the CBF4-CRF2 and RAV1-ERFs networks. The enriched pathways and involved TFs and DEGs are visualized using cytoscape. The genes shared by different pathways are indicated in gray. (D) The specific GO terms identified for CBF4-CRF2 and RAV1-ERFs networks. The enriched pathways and involved TFs and DEGs are shown.

For a given TF, the TF gene and its regulating targets generally have a high correlation relationship (Kuang et al. 2020). WGCNA can generate undirected weighted network with scale-free topology and discern such relationships (Zaidi et al. 2019, Kuang et al. 2020). To explore the possible target genes for identified TFs, we conducted WGCNA based on the RNA-seq results. According to the result, the highly correlated genes were enriched in the same modules (**Supplementary Fig. S6**). We focused on the gene modules involving the nine identified TFs and found that six out of the nine TFs could be successfully divided into two representative groups: the CBF4- and CRF2-dependent and the RAV1- and ERFs-dependent regulatory networks (**Fig. 4A**). In the first network, *CBF4* and *CRF2* function as hub genes, while, in the latter one, *RAV1, ERF1A, ERF104* and *ERF4* were detected as hub genes (**Fig. 4A**), suggesting that these TFs might function in cold response through at least two different signaling pathways. Although these co-expression genes were grouped together, their expression patterns in response to cold treatment were different (**Fig. 4A**). The TFs genes, as well as their possible target genes with significant changes in the expression (differentially expressed genes, DEGs), were selected to perform the canonical pathway enrichment. The results of GO analysis revealed that many of the DEGs from the two modules are involved in the same biological process and cellular component, such as response to abiotic stimulus and abscisic acid, flavonoid biosynthetic process and plasma membrane (**Fig. 4B**–**C**; Supplementary Dataset 2–3). Additionally, specific GO terms were also identified for the two gene modules. The DEGs from the CBF4 and CRF2 module were differentially enriched in cellular response to cytokinin stimulus, glutathione, glucose 6-posphate and glycerolipd metabolic process and transmembrane receptor activity and so on (**Fig. 4B**, **D**; Supplementary Dataset 2). In contrast, the DEGs from RAV1 and ERFs regulatory network were specifically enriched in GO terms involving phytohormones, such as ethylene biosynthetic process, ethylene-activated signaling pathway, salicylic acid and auxin metabolic process (**Fig. 4B**, **D**; Supplementary Dataset 3). Moreover, there is also a number of DEGs involved in the sucrose metabolic process (**Fig. 4D**). These results suggest that the identified TFs, which were clustered into two groups, may function in response to cold stress through different pathways.

### Overexpression of *VaRAV1* contributes to the cold tolerance of grape cells

**Fig. 5:**
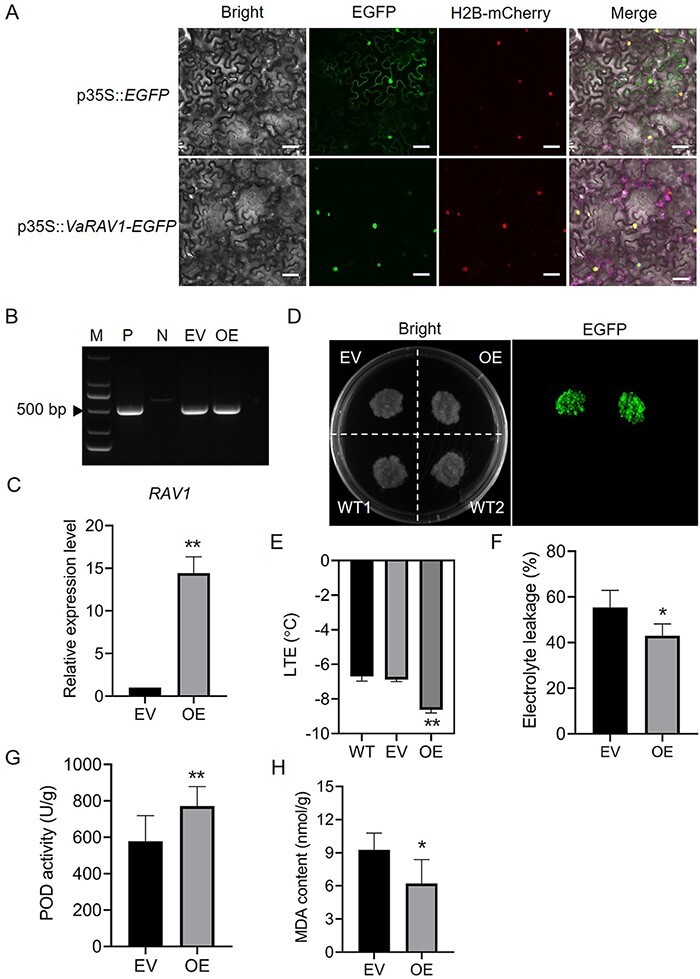
Overexpression of *VaRAV1* improves cold tolerance of grape cells. (A) Subcellular localization of VaRAV1. The coding sequence of *VaRAV1* fused to the N-terminal of *EGFP* gene was under the control of 35S promoter (p35S). The EGFP fluorescence generated by the p35S::*VaRAV1-EGFP* construct in epidermal cells of *Nicotiana benthamiana* leaves was detected using confocal laser scanning microscopy. The histone H2B-mCherry was used as an indicator of nucleus. Scale bars correspond to 200 μm. (B) PCR identification of exogenous T-DNAs using *EGFP*-specific primers in 41B grape cells. The plasmid of construct and wild-type cells were used as positive (P) and negative (N) controls, respectively. EV, grape cells transformed with empty vector; OE, VaRAV1-overexpressing cells. M, DNA marker. (C) Measurement of *RAV1* expression level in the OE cells. The expression of *RAV1* in the EV cells was set as 1, and the transcript abundance of *RAV1* in the OE cells relative to the EV was determined by qPCR. The data are mean values ±SD of three biological replicates. (D) Detection of EGFP signals in the EV and OE cells. WT, wild-type cells. (E) LTEs of the EV and OE cells. The data are mean values ±SD of five biological replicates. (F) Electrolyte leakage of the EV and OE cells after cold treatment. (G) POD activity in transgenic grape cells after cold treatment. (H) Measurement of MDA content in the EV and OE cells after cold treatment. Asterisks (*) and (**) indicate significant differences compared with the EV at *P* <0.05 and *P* <0.01 (Student’s *t*-test), respectively.

As mentioned above, nine TF genes, including the *CBF4*, were identified as candidate genes in response to cold stress. Given that the role of *CBFs* in cold response had been well characterized in many plant species, including grapevine (Xiao et al. 2006, 2008), we selected *RAV1, ERF1A* (up-regulated) and *CRF2* (down-regulated) as the candidate genes to test their functions in cold tolerance in grape. The coding sequences (CDSs) of the three genes were amplified from *V. amurensis*, respectively. Several nucleotide changes were observed between the amplified CDSs of the three genes and their reference sequences (**Supplementary Figs. S7–S9**). Subcellular localization assays revealed that the VaRAV1-EGFP fusion protein, as well as the VaERF1A-EGFP and VaCRF2-EGFP proteins, was exclusively localized in the nucleus, whereas the EGFP fluorescence was detected in both cytoplasm and nucleus (**Fig. 5A**; **Supplementary Figs. S10**–**S11**). To evaluate the functions of the three genes in cold tolerance, we overexpressed *VaRAV1, VaERF1A* and *VaCRF2* in 41B grape cells, respectively. After antibiotic-dependent selection, the kanamycin-resistant cells were first confirmed by PCR with *EGFP*-specific primers (**Fig. 5B**; **Supplementary Fig. S12**). The results of qPCR showed that the expression of *RAV1* in *VaRAV1*-overexpressing (OE) cells was almost 15-fold higher than that in 41B cells transformed with empty vector (EV; **Fig. 5C**). The detection of the EGFP fluorescence further confirmed the expression of VaRAV1-EGFP in the OE cells (**Fig. 5D**). However, the expression of *ERF1A* and *CRF2* in corresponding transgenic cells was not increased when compared with the EV cells (**Supplementary Fig. S12**), and the fluorescence of EGFP in these cells was weak (data not shown). We speculated that the low transformation rate of 41B cells resulted in the presence of plenty of untransformed cells, which affected the determination of gene expression. Thus, only the *VaRAV1*-OE (hereafter referred as OE) cells were used for subsequent analysis. The cold tolerance of the OE and EV cells was evaluated by the low temperature exotherms (LTEs) as described by Mills et al. (2006) and Sun et al. (2018). The LTEs of WT, EV and OE were −6.7, −6.9 and −8.6°C, respectively (**Fig. 5E**), suggesting that the overexpression of *VaRAV1* enhanced the cold tolerance of 41B cells. In addition, we conducted freezing treatment with the OE and EV cells. After exposure to freezing temperature (−4°C, 0.5 h), the number of apoptotic cells, which are stained by Trypan blue, was obviously increased (**Supplementary Fig. S13**). Although no much difference was observed in staining results between the EV and OE cells, the electrolyte leakage of the OE cells, however, was significantly lower than that of the EV cells after freezing treatment (**Fig. 5F**). Furthermore, the peroxidase (POD) activity was higher whereas the malondialdehyde (MDA) content, which serves as an indicator of ROS damage to membrane, was much lower in the OE cells (**Fig. 5G**–**H**). All these results indicate that the improved tolerance of the *VaRAV1*-OE cells to cold stress may be achieved partially by reducing membrane damage.

**Fig. 6:**
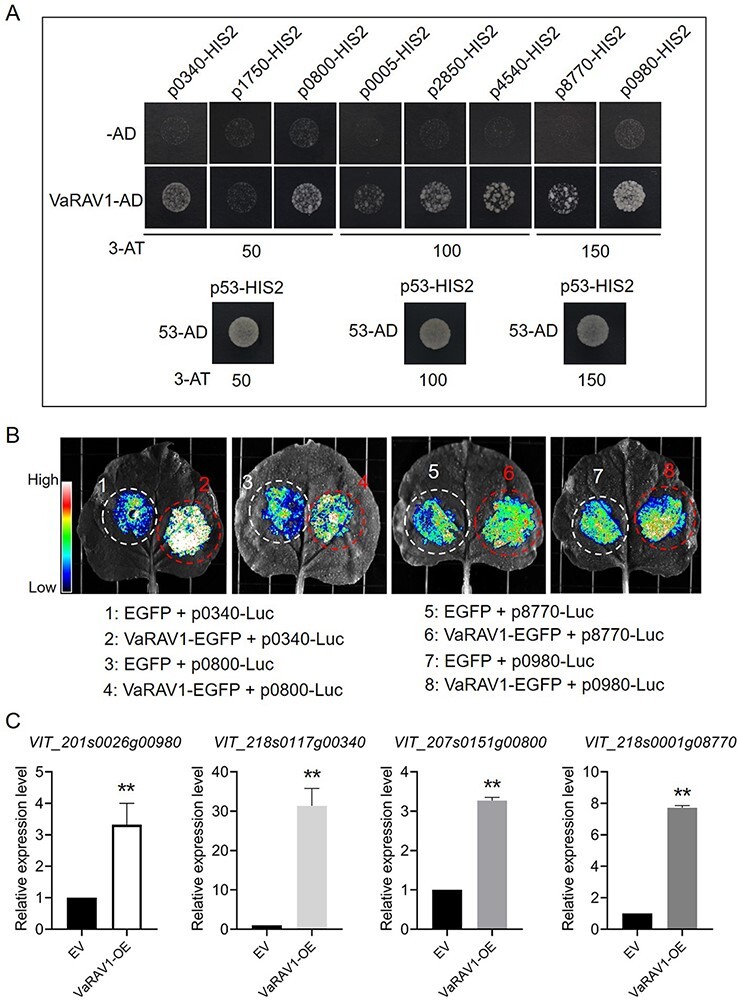
Detection of interactions between VaRAV1 and its target genes. (A) Yeast one-hybrid (Y1H) assay. The DNA sequences containing the predicted RAV1 motifs were synthesized and ligated into the bait vector pHIS2. The bait vectors and the prey vector harboring VaRAV1 fused to the GAL4 activation domain were introduced into the yeast strain Y187. Growth of transformed yeast cells was analyzed on SD/-Trp/-Leu/-His medium supplemented with 3-amino-1,2,4-triazole (3-AT). The p53HIS2 and pGADT-Rec2-53 were co-transformed into Y187 as positive control. For convenience, the last four numbers of gene IDs (**Supplementary Table S3**) were adopted for short. (B) Transient luciferase (Luc) activity assays. The promoters of the tested genes (*VIT_218s0117g00340, VIT_207s0151g00800, VIT_218s0001g08770* and *VIT_201s0026g00980*) were ligated to the pCAMBIA1302-Luc reporter vector. *Agrobacterium* cells containing the Luc reporter vectors and p35S::*VaRAV1-EGFP* construct were co-infiltrated into *N. benthamiana* leaves. The possible interactions between VaRAV1 and tested genes were evaluated by Luc fluorescence intensities. (C) The expression profiles of *VIT_218s0117g00340, VIT_207s0151g00800, VIT_218s0001g08770* and *VIT_201s0026g00980* in the *VaRAV1*-OE cells. The expression of the four genes in the OE cells relative to the EV was measured by qPCR. Significant difference was determined by Student’s *t*-test. **P < 0.01.

To investigate a possible molecular mechanism by which VaRAV1 improves cold tolerance, we identified the possible target genes for VaRAV1. As mentioned above, the gene co-expression network was established using WGCNA (**Fig. 4A**; **Supplementary Fig. S6**). However, many false-positive connections may be included in the network given that the network was developed just based on expression analysis (Kuang et al. 2020). To reduce the false-positive connection rate, the DEGs co-expressed with RAV1 were used for the analysis of motif enrichment. The TF motifs are highly conserved in plants, and the presence of motifs in the promoters of the target genes indicates the functional relevance of corresponding TFs (Franco-Zorrilla et al. 2014, Lai et al. 2019). Eight genes containing RAV1 motifs in their promoters were therefore identified for subsequent experiments (**Supplementary Table S3**). The promoter fragments of these genes containing RAV1 motifs were synthesized as the bait sequences (**Supplementary Table S1**), while VaRAV1 fused with the trans-activating domain (AD) was used as the prey. Y1H assay showed that the yeast cells containing p0340-HIS2 (for *VIT_218s0117g00340*), p0800-HIS2 (for *VIT_207s0151g00800*), p2850-HIS2 (for *VIT_217s0000g02850*), p4540-HIS2 (for *VIT_211s0016g04540*), p8770-HIS2 (for *VIT_218s0001g08770*) or p0980-HIS2 (for *VIT_201s0026g00980*) grew normally on medium supplemented with 3-AT, indicating that VaRAV1 could bind to the promoters of these genes (**Fig. 6A**). We then amplified the promoters of these six genes from *V. amurensis*, and only five promoters were successfully cloned, with the exception being *VIT_211s0016g04540*, whose promoter was totally different from the reference. Notably, a miRNA coding gene (ENSRNA049996455) was found to localize in the gene body of *VIT_217s0000g02850*, and the emergence of a premature stop codon results in the early translation termination (**Supplementary Fig. S14**). We therefore reasoned that the truncated protein of VIT_217s0000g02850 may not exhibit normal function, and this gene was omitted from our analysis. Finally, the amplified promoters of *VIT_218s0117g00340, VIT_207s0151g00800, VIT_218s0001g08770* and *VIT_201s0026g00980* were used for transient Luc activity assays (**Supplementary Fig. S15**), and the results showed that the expression of the Luc reporter gene was promoted by VaRAV1 (**Fig. 6B**), suggesting that VaRAV1 might positively regulate the expression of *VIT_218s0117g00340, VIT_207s0151g00800, VIT_218s0001g08770* and *VIT_201s0026g00980*. To test this hypothesis, we investigated the expression profiles of these four genes in the *VaRAV1*-OE cells. The qPCR results showed that all of the four genes were significantly up-regulated in the *VaRAV1*-OE cells (**Fig. 6C**), which is consistent with the results of transient Luc assays and the RNA-seq (**Fig. 6B**; **Supplementary Table S3**).

## Discussion

Cold-responsive genes can be divided into two groups: early-response genes and late-response genes (Schade et al. 2004). The early-response genes are generally observed to be involved in transcriptional regulation in plants after exposure to cold (Shinozaki and Yamaguchi-Shinozaki 1997, Thomashow 1999). CBFs are key TFs that function in cold acclimation, and CBF-dependent signaling had been well characterized during cold signal transduction. Various TFs that regulate the expression of *CBFs* had been identified in the past two decades (reviewed in Shi et al. 2018). However, in addition to the CBFs, the initial events involved in transcriptional regulation in response to cold stress in plants are still largely unknown. In the present study, we attempted to conduct ATAC-seq assay, combined with RNA-seq results, to identify novel TFs that may function in early cold response in grapevine.

ATAC-seq has emerged as a powerful method to study gene regulatory networks in model plants (Wilkins et al. 2016, Lu et al. 2017, Maher et al. 2018, Sijacic et al. 2018, Potter et al. 2018), and specific methods, such as FANS (fluorescence-activated nuclei sorting) and INTACT (isolation of nuclei tagged in specific cell types), had been developed to remove cell walls and intracellular organelles during nuclei purification (Lu et al. 2017, Bajic et al. 2017, Potter et al. 2018, Maher et al. 2018, Sijacic et al. 2018). However, the purification of nuclei is not essential for the implementation of ATAC-seq, and the data using crude nuclei are comparable to that using INTACT-purified nuclei in *Arabidopsis* (Lu et al. 2017, Maher et al. 2018). Here, we performed ATAC-seq effectively using crudely purified nuclei to identify accessible *cis*-regulatory regions in *V. amurensis* after cold treatment, and nine TFs, including CBF4, were identified as the candidates involved in early cold response (2 h) in grapevine (**Fig. 3B**). Intriguingly, among the nine identified TFs, eight belong to the AP2/ERF superfamily except MYB108 (**Fig. 3B**; Supplementary Dataset 1). AP2/ERF superfamily includes AP2, ERF and RAV families. ERF family can be further divided into two subfamilies, namely ERF and CBF/DREB (Nakano et al. 2006). The role of CBF/DREB subfamilies in cold acclimation had been widely demonstrated, whereas the function of ERF family in plant cold tolerance remains less studied. Interestingly, the GO analysis of RAV1 and ERFs regulatory network revealed a role for ethylene signaling pathway in response to cold stress (**Fig. 4B**, **D**). Recently, a few reports suggested that ERFs contribute to cold tolerance in plants (Tian et al. 2011, Cheng et al. 2013, Bolt et al. 2017, Zhai et al. 2017). In grapevine, the VaERF057 and VaERF092 were found to improve cold tolerance (Sun et al. 2016, 2019). Actually, the expression of *ERFs* identified in this work, particularly the *ERF104* and *ERF1A*, was strongly induced by cold treatment (**Fig. 3B**). These *ERFs* could serve as good candidates for further study. As the members of ERF family, the expression of the *CRF2* and *ESE3*, however, were repressed by cold treatment (**Fig. 3B**), suggesting that the two TFs might function as negative regulators during the cold response in grapevine. Moreover, the *CRF2* was found to be in the same group with *CBF4* (**Fig. 4**), which encodes a positive regulator of cold tolerance. These results indicate that different TFs may cooperate to regulate the expression of downstream genes. Additionally, enriched GO terms for the identified TFs and their DEGs turned out to be involved in multiple physiological and biochemical pathways, suggesting that the signaling in response to cold stress in grapevine is complicated, and CBF4 and ERFs may participate in cold response through the common or their own regulatory pathways (**Fig. 4**).

In the present study, three TFs genes, among which the *RAV1* and *ERF1A* were induced, while the *CRF2* was repressed by cold stress, were amplified from *V. amurensis*. The cloned sequences exhibited nucleotide changes compared with their reference sequences, which results in substitutions or insertions of amino acids (less than 5 aa; **Supplementary Figs. S7–S9**). The possible effect of aa changes on TFs functions was not investigated in this study, and more evidence is needed to confirm whether these changes were conserved in grape across different species. Because the *VaERF1A* and *VaCRF2* were not overexpressed in grape cells after transformation as expected (**Supplementary Fig. S12**), only *VaRAV1* was further studied to verify its function in cold tolerance. In *Arabidopsis*, RAV1 was known as a negative regulator of plant growth (Woo et al. 2010, Feng et al. 2014). The overexpression of *Arabidopsis RAV1* increased the sensitivity of transgenic plants to salt and drought stress (Fu et al. 2014, Sengupta et al. 2020). Nevertheless, the expression of *RAV1* was rapidly induced in parallel with *CBF1, 2*, and *3* by low temperature in *Arabidopsis* (Fowler et al. 2005). The involvement of RAV1 in *Galegae orientalis* cold tolerance was suggested by Chen et al. (2009). However, the evidence for the role of RAV1 in cold tolerance is still scarce. The overexpression of *VaRAV1* resulted in a lower value of LTE of grape cells (**Fig. 5E**), and the electrolyte leakage of the *VaRAV1*-OE cells were much lower than that of the control (EV) cells (**Fig. 5F**). High POD activity and low MDA content were also observed in the *VaRAV1*-OE cells (**Fig. 5G**, **H**). These results provide evidence for the function of VaRAV1 in cold tolerance in grape. In addition, four candidate genes, *VIT_218s0117g00340, VIT_207s0151g00800 VIT_218s0001g08770* and *VIT_201s0026g00980* were positively regulated by VaRAV1 (**Fig. 6**). Among the four genes, *VIT_218s0117g00340* encodes the TCP8 TF, while *VIT_207s0151g00800* and *VIT_218s0001g08770* encode MARD1 (MEDIATOR OF ABA-REGULATED DORMANCY 1)-like and PHLOEM PROTEIN 2-LIKE A10 protein, respectively (**Supplementary Table S3**). The TCP proteins were generally found to be associated with plant growth and development (Lopez et al. 2015, Leng et al. 2019). The involvement of TCP proteins, as well as MARD1 and PHLOEM PROTEIN, in cold tolerance remains less investigated. Intriguingly, a protein of galacturonosyltransferase (GAUT)-like 9 is encoded by the *VIT_201s0026g00980* gene (**Supplementary Table S3**). GAUT is an enzyme that synthesizes homogalacturonan (HG), which is the most abundant pectic polysaccharide in primary cell wall and the middle lamella (Mohnen 2008, Atmodjo et al. 2011). The structure and composition of the plant primary cell wall are important in determining freezing tolerance of plants (Panter et al. 2019). We speculated that VaRAV1 might contribute to the cold tolerance by affecting the composition of plant cell wall through the positive regulation of *VIT_201s0026g00980*.

Taken together, the present work provides us new insights into transcriptional regulations involved in early cold response in *V. amurensis*. The ethylene-activated signaling pathway probably plays a significant role in cold response, and the CBF-dependent pathway, as well as the other signaling pathways involving different physiological and biochemical processes, is also important in response to cold stress. Our experimental results revealed that VaRAV1 could enhance the tolerance of grape cells to cold stress, demonstrating the robustness of our approach. The other ERFs and the TF MYB108 identified here could serve as pertinent candidates for further study. More importantly, our results suggest that ATAC-seq, combined with RNA-seq, is suitable for the identification of novel TFs in grapevine, and this approach can be adopted as a useful tool for functional genomics research.

## Materials and Methods

### Plant materials and cold treatment

The Chinese wild-growing *V. amurensis* seedlings were grown in the grapevine germplasm resources orchard at the Institute of Botany, Chinese Academy of Sciences, Beijing. One-month-old *in vitro* subcultured plants developing from *V. amurensis* cuttings were used for cold treatment as previously described (Xu et al. 2014) with some modifications. Around 100 *in vitro* plants with uniform growth status were transferred to a prechilled incubator (LRH-200-GD, Taihong Scientific Instrument Co., Ltd, Shaoguan, China) set to 4°C and constant light (approximately 100 μmol m^−2^ s^−1^) for cold treatment. The upper two leaves of five individual plants were harvested and pooled as a biological replicate at different time points (2, 4, 8, 12 and 24 h). Untreated plants cultured at 26°C under the same light condition were sampled as the control (0 h). The samples at each time point were prepared in triple for biological replicates and immediately used for electrolyte leakage analysis or frozen in liquid nitrogen and stored at −80°C.

### ATAC-seq library preparation

The grapevine leaves collected at 0 and 2 h of cold treatment were ground into powder with liquid nitrogen, and intact nuclei were isolated as previously described (Wilkins et al. 2016). The isolated nuclei were immediately used for transposase tagmentation. The transposase tagmentation was performed as previously described (Sijacic et al. 2018) with some modifications. In brief, the purified nuclei were resuspended in a 50-μL transposase integration reaction using Nextera kit, and the transposition reaction was incubated at 37°C for 30 min. Tagmented DNA was immediately purified using the Qiagen MiniElute PCR Purification Kit and eluted in 10 μL of elution buffer. Then the DNA fragments were amplified using high-fidelity PCR mix (NEB) with custom-barcoded primers for 5–10 cycles. The amplified ATAC-seq libraries were purified using AMPure XP beads (Beckman Coulter) and sequenced as paired-end 50-bp reads on an Illumina HiSeaq2500 instrument. The ATAC-seq and subsequent bioinformatics analysis were performed by Shanghai Jiayin Biotechnology Co., Ltd.

### ATAC-seq data processing

Raw sequence reads were initially processed by FastQC (Babraham Institute, Cambridge) for quality control, and adaptor sequences and poor-quality reads were removed by using Cutadapt. Quality-filtered reads were then mapped to grape 12X genome (version 2.1) using BWA (Langmead and Salzberg 2012). Only uniquely mapped reads were kept, and duplicates were removed using Samtools. Mapped reads in .sam format were converted to .bam format using Samtools (Li et al. 2009) for peak calling. MACS2 (Zhang et al. 2008) was used to call peaks with an initial threshold *q*-value of 0.05 as cutoff, and the peaks were identified with parameters callpeak -nomodel -shift 0 -extsize 200. The called peaks were referred as transposase hypersensitive sites or THSs. For the visualization of read count data, the .bam files were first converted to bigwig files, and genome browser images were made using the Integrative Genomics Viewer (IGV) tools (Thorvaldsdottir et al. 2013).

### Genomic distribution of THSs

The called peaks or THSs that were found in at least two biological replicates were mapped to genome features to determine their distributions. The distribution of THSs relative to genomic features was assessed with ‘upstream’ regions set as 3000-bp upstream of the annotated TSS and ‘downstream’ regions set as 1000-bp downstream of the TES by using the PAVIS web tool (Huang et al. 2013). Besides, for each ATAC-seq dataset, all the THSs were assigned to the closest transcription start sites regardless of the positions (upstream or downstream) from the THSs to identify THS-proximal genes by using the ‘TSS’ function of the PeakAnnotator 1.4 program (Salmon-Divon et al. 2010).

### Differential THSs for motif analysis

To identify specific open accessible regions after cold treatment, the differential accessible peaks were screened and analyzed. The peaks that were found in three biological replicates of each sample were calculated and merged as a file using the bedtools software. The counts of the reads were determined for each sample using bedtools multicov. Differential accessible peaks were determined using DESeq2, and those regions identified with a value of log2FC ≥1 (positively enriched) or ≤−1 (negatively enriched) and a *P* value <0.05 were considered as specific accessible peaks. These peaks were then used for motif analysis with the HOMER’s FindMotifGenome.pl tool. The peak files and grape genome fasta files were adopted as input files. The extracted DNA sequences were compared with known motif database (JASPAR, http://jaspar.genereg.net/) to obtain overrepresented motifs.

### Identification of motifs within gene promoters

To identify motifs within promoters of genes of interest, the 2-kb upstream sequences of genes were downloaded as the promoters from the grape genome (http://genomes.cribi.unipd.it/grape/index.php), and the motif profiles used for motif occurrence identification were downloaded from JASPAR and HOMER databases using gene names or IDs as the inputs. The motif occurrences were identified using the function of motif scanning of FIMO (http://meme-suite.org/tools/fimo; Grant et al. 2011). Those motif occurrences with a *P* value <0.0001 were considered significant.

### WGCNA and GO analysis

The WGCNA was conducted with RNA-seq data using R package to develop gene co-expression networks. The log2(FPKM of each treatment +1) was used as input, and soft-thresholding was set to 6. The GO analysis was conducted using the agriGO v2.0 with default parameters (Tian et al. 2017). The GO terms with a corrected *P* value of (false discovery rate, FDR) <0.05 were considered significant. The co-expression networks and enriched GO terms were visualized using the cytoscape software.

### RNA-seq and quantitative real-time PCR (qPCR)

Total RNA was extracted using Spectrum Plant Total RNA Kit (Sigma-Aldrich) according to the manufacturer’s protocol. The prepared RNA samples were used for RNA-seq analysis. The transcriptome library was prepared using NEBNext Ultra RNA Library Prep Kit for Illumina following the manufacturer’s instruction. Briefly, messenger RNA was purified from total RNA using oligo (dT) beads, and then fragmented with fragmentation buffer. Double-stranded cDNA was synthesized with the SuperScript double-stranded cDNA synthesis kit (Invitrogen) using random hexamer primers. After the adenylation of 3ʹends of DNA fragments, NEBNext Adaptor with hairpin loop structure were added to prepare for hybridization. The libraries were purified with AMPure XP system (Beckman Coulter) to select cDNA fragments of 150–200 bp, followed by PCR amplified using Phusion High-Fidelity DNA polymerase (NEB). Finally, the quality of libraries was assessed on the Agilent Bioanalyzer 2100 system and sequenced with the Illumina Hiseq 4000 (125/150-bp paired-end reads). The raw reads were first processed with FastQC for quality control, and sequences with adaptor and poor-quality reads were removed. High-quality reads were then mapped to grape 12X genome (version 2.1) using STAR. HTSeq was used to calculate the numbers of unique reads. Differentially expressed genes were analyzed using the DESeq R package (*P *< 0.05). RNA-seq and data analysis were conducted by Shanghai Jiayin Biotechnology Co., Ltd.

For qPCR, the cDNA was synthesized from 1 μg of total RNA with the HiScript II Q RT SuperMix Kit (Vazyme). qPCR was performed using the CFX Manager system (BioRad) and SYBR Green Master Mix (Vazyme). The *Actin* (GenBank accession no.100232866) and *GAPDH* (GenBank accession no.100233024) genes were used as internal controls. Primers used for qPCR are available in **Supplementary Table S1**. The obtained data were calculated using 2^-ΔΔCT^ method (Livak and Schmittgen 2001), and the test of significance was performed using unpaired Student’s *t*-test.

### Cloning of gene CDSs and promoters and construction of vectors

The full-length CDSs of *RAV1, ERF1A* and *CRF2* were amplified from the cDNA libraries of *V. amurensis* by PCR with their specific primers using the KOD-Plus-Neo Kit (TOYOBO). Similarly, the promoters of *VIT_217s0000g02850, VIT_218s0117g00340, VIT_207s0151g00800, VIT_218s0001g08770* and *VIT_201s0026g00980* were amplified using genomic DNA as the template. The amplified products were cloned into the pLB cloning vector (TIANGEN) for sequencing.

To develop the vector for overexpression and subcellular localization, the sequences of *VaRAV1, VaERF1A* and *VaCRF2* without stop codons were amplified from the pLB vectors using the primers sets RAV1-2300, ERF1A-2300 and CRF2-2300, respectively, and inserted into the *Bam*HI-digested pCAMBIA2300-EGFP vector via homologous recombination (HR) using the ClonExpress II One Step Cloning Kit (Vazyme). The fusion genes are driven by CaMV 35S promoter. To generate Luc reporter constructs, the promoters were amplified from the pLB vectors using their own specific primers sets and ligated into the *Kpn*I-digested pCAMBIA1302-Luc vector by HR. To produce the vectors for Y1H assay, *VaRAV1* was cloned into the pGADT7 vector (Clontech) through HR. A 30–34-bp DNA sequence of gene promoter containing the RAV1 motif(s) and its complementary sequence were synthesized as primers for each gene. The forward and reverse primers were mixed at a ratio of 1:1, and double-stranded DNA fragment was formed through denaturation and annealing steps. The DNA fragment was ligated into the pHIS2 (Clontech) vector via *Eco*RI and *Sac*I sites. All the primers are provided in **Supplementary Table S1**.

### Identification of candidate target genes for VaRAV1

To identify candidate target genes for VaRAV1, all the DEGs co-expressed with VaRAV1 were used for motif analysis as described above. The DEGs containing RAV1 motif(s) in their promoters were selected for subsequent Y1H and transient Luc assays.

### Transient Luc assay

For transient Luc expression assay, the Luc reporter vectors were introduced into the *Agrobacterium* strain GV3101, and the overnight cultured *Agrobacterium* cells were collected and resuspended in the injection buffer (10 mM MgCl_2_, 10 mM MES and 1 mM Acetosyringone) to a final OD600 of 0.6. The bacterial cells were mixed at a ratio of 5:1 (*VaRAV1*: Luc). The mixtures were incubated at room temperature for 2 h before injection. The 7-week-old *Nicotiana benthamiana* leaves were used for injection. The Luc fluorescence was measured by luminescence intensity using CCD camera (Tanon 5200).

### Y1H assay

Plasmids of bait vector and prey vector were introduced into the yeast strain Y187 (Clontech) and cultured on SD/-Leu/-Trp medium. Transformants were picked and cultured in liquid SD/-Leu/-Trp medium for 12–16 h and then collected by centrifugation. The yeast cells were resuspended in sterilized water and diluted to an OD600 of 0.1. Around 4 μL of suspension was spotted on SD/-Leu/-Trp/-His medium with optimal concentration of 3-amino-1,2,4-triazole (3-AT). The plates were incubated at 30°C for 2–3 d. The yeast cells containing the recombinant pHIS2 vector were spotted on SD/-Trp/-His medium to identify the optimal 3-AT concentration for each vector.

### Subcellular localization

The *N. benthamiana* leaves were infiltrated with *Agrobacterium* cells containing 35S::*VaRAV1-EGFP*, 35S::*VaERF1A-EGFP* and 35S::*VaCRF2-EGFP* constructs, respectively, and the bacterial cells harboring the histone H2B-mCherry expression vector were co-infiltrated at a ratio of 1:1. The fluorescence was detected using Leica TCS SP8 confocal laser scanning microscopy. The experiment was repeated three times.

### Transformation of 41B cells and detection of EGFP

For the transformation of 41B cells, the developed overexpression constructs were introduced into the *Agrobacterium* strain EHA105. The 41B (*V. vinifera* × *V. berlandieri*) grape cells were used as explants for *Agrobacterium*-mediated transformation. The empty vector 35S::*EGFP* was used as a control. The transformation was performed as previously described (Ren et al. 2019). After transformation, the 41B cells were cultured on kanamycin-containing medium until the generation of resistant cells. Transgenic cells were identified by PCR with *EGFP*-specific primers (**Supplementary Table S1**) and also by qPCR and EGFP fluorescence detection. EGFP signal of 41B cells was detected using CCD camera (Tanon 5200).

### Physiological analyses

The *V. amurensis* leaves after cold treatment were directly used for electrolyte leakage assay. For the transgenic 41B cells, the cells were cultured on solid medium at 26°C for 15 d and then transferred to 4°C for another 3 d. Freezing treatment began at 4°C, the temperature dropped by 1°C per minute until reaching the temperature of −4°C, and the plates were kept at −4°C for 0.5 h. After treatment, the plates were incubated at 4°C for 12 h and then cultured at 26°C for 1 d. The treatment was performed under dark conditions. The grape cells after freezing treatment were used for Trypan blue (ScienCell) staining, electrolyte leakage assay and measurement of POD (peroxidase) activity and MDA (malondialdehyde) content.

Electrolyte leakage assay was performed as described previously (Li et al. 2017) with modifications. Shortly, six leaf disks with a diameter of 10 mm or 0.1 g of 41B cells were placed in 10 mL tubes containing 4 mL deionized water (S0), shaken at 180 rpm for 20 min and measured S1. Then the samples were boiled for another 20 min, shaken at room temperature for 1 h and detected S2. The value (S1-S0)/(S2-S0) was calculated as electrolyte leakage. The POD activity and MDA content were measured using POD and MDA isolation kits (Solarbio) according to the manufacturer’s protocols. All the experiments were performed with as least three biological and three technical replicates.

### Measurement of L TEs

The LTEs were commonly used to evaluate the cold tolerance of grape calli (Sun et al. 2018, 2019). The cold tolerance of the transgenic 41B cells was evaluated by measuring the LTEs as described by Sun et al. (2018). Five biological replicates were used for the measurement.

## Supplementary Material

pcab115_Supp

## Data Availability

The data underlying this article are available in NCBI Gene Expression Omnibus (GEO) database at https://www.ncbi.nlm.nih.gov/geo/ and can be accessed with GSE166247 (ATAC-seq) and GSE166247 (RNA-seq), respectively.
