# Supplementary material for: Characterization of Chromatin Accessibility and Gene Expression upon Cold Stress Reveals that the RAV1 Transcription Factor Functions in Cold Response in Vitis Amurensis
Source: Plant Cell Physiol. 2021 Jul 19;62(10):1615–29. doi: 10.1093/pcp/pcab115 (PMC8643690; doi:10.1093/pcp/pcab115)
Supplement: pcab115_Supp [file pcab115_supp.zip › pcp-2021-e-00082-File008.pdf]

Supplementary Figures and Tables

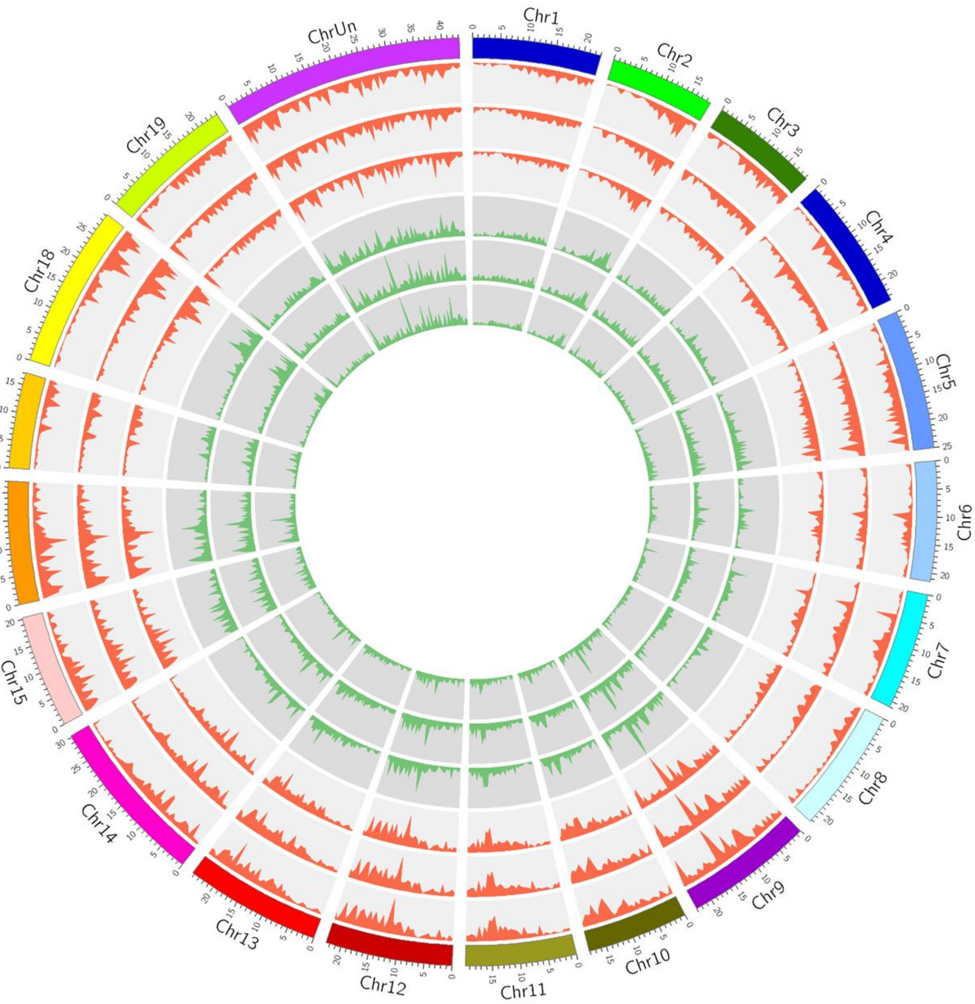

Fig. S1. Distribution of ATAC-seq reads relative to grape chromosomes. ATAC-seq reads were mapped to the grape genome, and distribution of the reads from three biological replicates at 0 h and 2 h are indicated in orange and green, respectively. The outmost regions in different colors represent grape chromosomes.

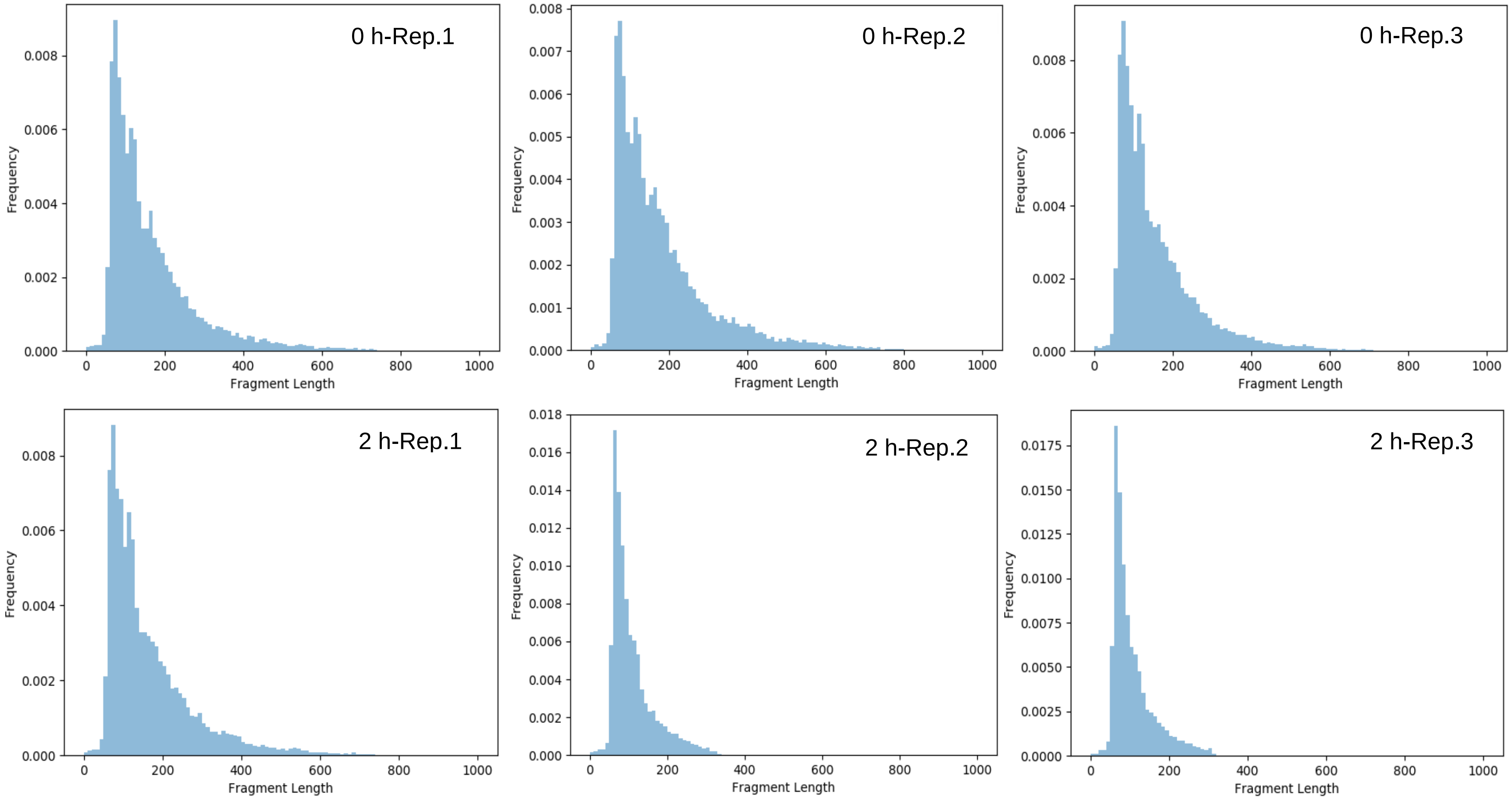

Fig. S2. Fragment length distributions of reads from six ATAC-seq libraries.

A

Differential peaks/THSs identified after cold treatment		
No. of positively enriched THSs at 2 h	No. of negatively enriched THSs at 2 h	Percentage of differential THSs
1376	189	11.7%

B

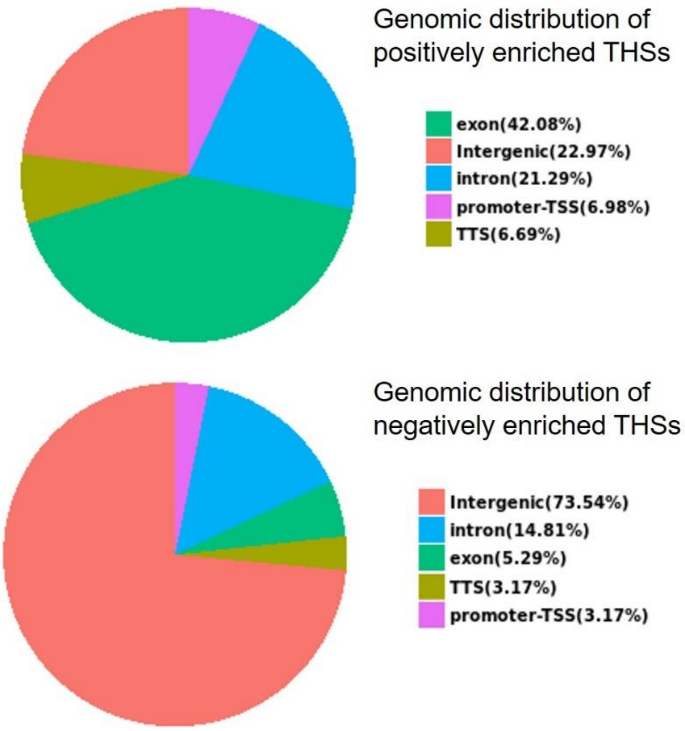

Fig. S3. Number and genomic distribution of differentially enriched THSs after cold treatment. (A) Number of differential THSs identified after cold treatment. (B) Genomic distribution of positively enriched THSs after cold treatment. (C) Genomic distribution of negatively enriched THSs after cold treatment.

Rank	Motif	Name	P-value	log P-pvalue	q-value (Benjamini)	# Target Sequences with Motif	% of Targets Sequences with Motif
1	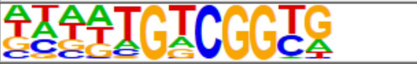	AT1G71450(AP2EREBP)/col-AT1G71450-DAP-Seq(GSE60143)/Homer	1e-8	-1.865e+01	0.0000	314.0	22.82%
2	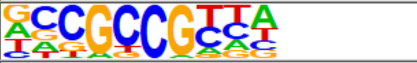	CRF10(AP2EREBP)/col100-CRF10-DAP-Seq(GSE60143)/Homer	1e-6	-1.431e+01	0.0003	210.0	15.26%
3	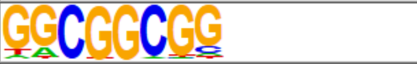	AT1G28160(AP2EREBP)/colamp-AT1G28160-DAP-Seq(GSE60143)/Homer	1e-6	-1.426e+01	0.0003	245.0	17.81%
4	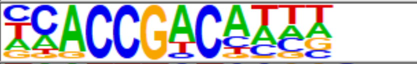	DEAR2(AP2EREBP)/colamp-DEAR2-DAP-Seq(GSE60143)/Homer	1e-6	-1.393e+01	0.0003	216.0	15.70%
5	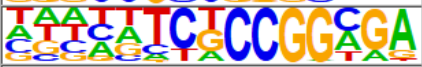	AT5G05550(Trihelix)/col-AT5G05550-DAP-Seq(GSE60143)/Homer	1e-5	-1.203e+01	0.0012	338.0	24.56%
6	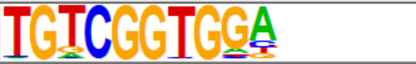	At1g77640(AP2EREBP)/col-At1g77640-DAP-Seq(GSE60143)/Homer	1e-4	-1.137e+01	0.0019	56.0	4.07%
7	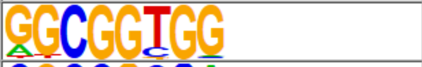	AT3G57600(AP2EREBP)/col-AT3G57600-DAP-Seq(GSE60143)/Homer	1e-4	-1.132e+01	0.0019	108.0	7.85%
8	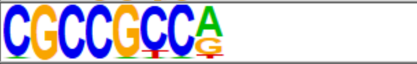	CRF4(AP2EREBP)/colamp-CRF4-DAP-Seq(GSE60143)/Homer	1e-4	-1.099e+01	0.0021	110.0	7.99%
9	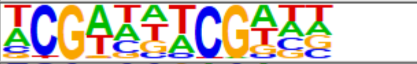	AT5G22990(C2H2)/col-AT5G22990-DAP-Seq(GSE60143)/Homer	1e-4	-1.084e+01	0.0021	80.0	5.81%
10	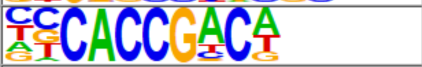	AT1G44830(AP2EREBP)/col-AT1G44830-DAP-Seq(GSE60143)/Homer	1e-4	-1.069e+01	0.0022	72.0	5.23%
11	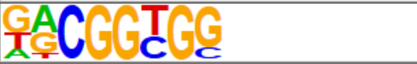	ESE3(AP2EREBP)/col-ESE3-DAP-Seq(GSE60143)/Homer	1e-4	-1.053e+01	0.0024	196.0	14.24%
12	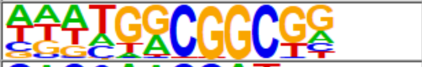	ERF4(AP2EREBP)/colamp-ERF4-DAP-Seq(GSE60143)/Homer	1e-4	-1.034e+01	0.0026	192.0	13.95%
13	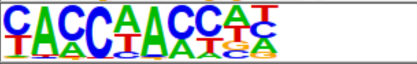	MYB83(MYB)/colamp-MYB83-DAP-Seq(GSE60143)/Homer	1e-4	-1.032e+01	0.0026	342.0	24.85%
14	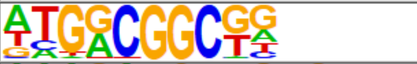	ERF7(AP2EREBP)/col-ERF7-DAP-Seq(GSE60143)/Homer	1e-4	-1.029e+01	0.0026	216.0	15.70%
15	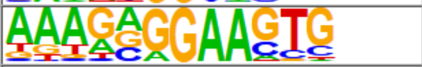	SpiB(ETS)/OCILY3-SPIB-ChIP-Seq(GSE56857)/Homer	1e-4	-1.018e+01	0.0026	53.0	3.85%
16	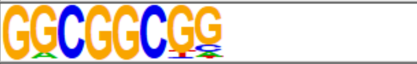	ERF104(AP2EREBP)/col-ERF104-DAP-Seq(GSE60143)/Homer	1e-4	-1.012e+01	0.0026	144.0	10.47%
17	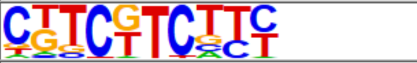	Unknown4/Arabidopsis-Promoters /Homer	1e-3	-9.099e+00	0.0064	306.0	22.24%
18	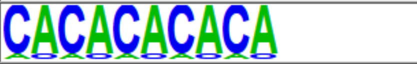	SeqBias: CA-repeat	1e-3	-8.990e+00	0.0068	771.0	56.03%
19	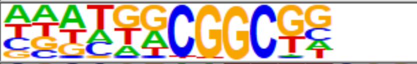	RAP26(AP2EREBP)/colamp-RAP26-DAP-Seq(GSE60143)/Homer	1e-3	-8.817e+00	0.0076	201.0	14.61%
20	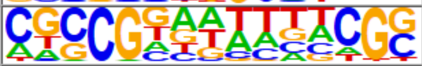	LOB(LOBAS2)/col-LOB-DAP-Seq(GSE60143)/Homer	1e-3	-8.759e+00	0.0077	62.0	4.51%
21	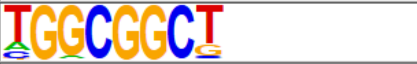	ERF105(AP2EREBP)/colamp-ERF105-DAP-Seq(GSE60143)/Homer	1e-3	-8.501e+00	0.0095	190.0	13.81%
22	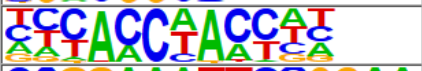	MYB13(MYB)/col-MYB13-DAP-Seq(GSE60143)/Homer	1e-3	-8.184e+00	0.0124	143.0	10.39%
23	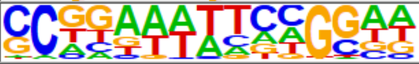	LBD19(LOBAS2)/colamp-LBD19-DAP-Seq(GSE60143)/Homer	1e-3	-7.922e+00	0.0154	312.0	22.67%
24	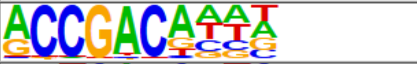	AT1G77200(AP2EREBP)/colamp-AT1G77200-DAP-Seq(GSE60143)/Homer	1e-3	-7.796e+00	0.0167	160.0	11.63%
25	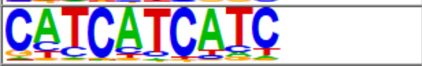	ZML2(C2C2gata)/col-ZML2-DAP-Seq(GSE60143)/Homer	1e-3	-7.679e+00	0.0181	87.0	6.32%
26	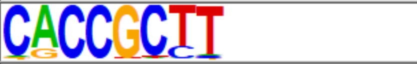	At5g18450(AP2EREBP)/col-At5g18450-DAP-Seq(GSE60143)/Homer	1e-3	-7.556e+00	0.0196	238.0	17.30%
27	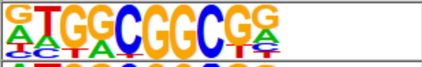	ERF10(AP2EREBP)/col-ERF10-DAP-Seq(GSE60143)/Homer	1e-3	-7.388e+00	0.0224	114.0	8.28%
28	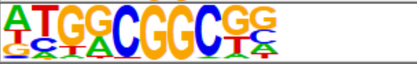	ERF3(AP2EREBP)/colamp-ERF3-DAP-Seq(GSE60143)/Homer	1e-3	-7.033e+00	0.0307	113.0	8.21%
29	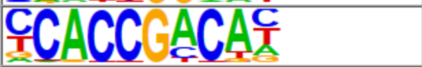	DREB26(AP2EREBP)/col-DREB26-DAP-Seq(GSE60143)/Homer	1e-3	-6.934e+00	0.0328	55.0	4.00%
30	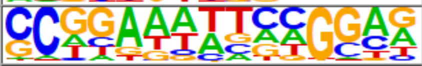	ASL18(LOBAS2)/colamp-ASL18-DAP-Seq(GSE60143)/Homer	1e-2	-6.905e+00	0.0328	264.0	19.19%

31	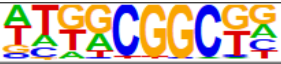	At2g33710(AP2EREBP)/colamp-At2g33710-DAP-Seq(GSE60143)/Homer	1e-2	-6.861e+00	0.0330	278.0	20.20%
32	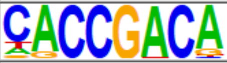	ERF38(AP2EREBP)/col-ERF38-DAP-Seq(GSE60143)/Homer	1e-2	-6.861e+00	0.0330	98.0	7.12%
33	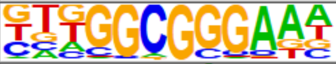	E2F3(E2F)/MEF-E2F3-ChIP-Seq(GSE71376)/Homer	1e-2	-6.857e+00	0.0330	68.0	4.94%
34	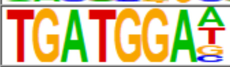	HAP3(CCAATHAP3)/col-HAP3-DAP-Seq(GSE60143)/Homer	1e-2	-6.805e+00	0.0330	97.0	7.05%
35	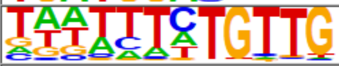	RAV1(RAV)/colamp-RAV1-DAP-Seq(GSE60143)/Homer	1e-2	-6.768e+00	0.0330	156.0	11.34%
36	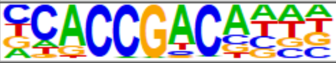	At1g22810(AP2EREBP)/colamp-At1g22810-DAP-Seq(GSE60143)/Homer	1e-2	-6.744e+00	0.0330	99.0	7.19%
37	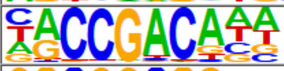	At4g16750(AP2EREBP)/col-At4g16750-DAP-Seq(GSE60143)/Homer	1e-2	-6.487e+00	0.0402	155.0	11.26%
38	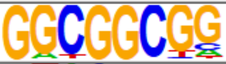	ESE1(AP2EREBP)/col-ESE1-DAP-Seq(GSE60143)/Homer	1e-2	-6.463e+00	0.0402	106.0	7.70%
39	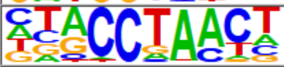	MYB62(MYB)/colamp-MYB62-DAP-Seq(GSE60143)/Homer	1e-2	-6.451e+00	0.0402	205.0	14.90%
40	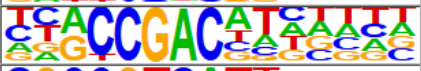	CBF2(AP2EREBP)/colamp-CBF2-DAP-Seq(GSE60143)/Homer	1e-2	-6.406e+00	0.0403	108.0	7.85%
41	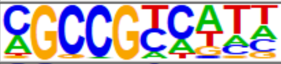	ERF8(AP2EREBP)/colamp-ERF8-DAP-Seq(GSE60143)/Homer	1e-2	-6.369e+00	0.0408	183.0	13.30%
42	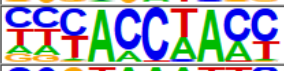	MYB58(MYB)/colamp-MYB58-DAP-Seq(GSE60143)/Homer	1e-2	-6.308e+00	0.0423	182.0	13.23%
43	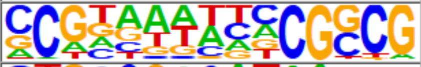	AS2(LOBAS2)/col-AS2-DAP-Seq(GSE60143)/Homer	1e-2	-6.282e+00	0.0424	25.0	1.82%
44	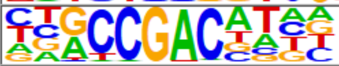	CBF3(AP2EREBP)/colamp-CBF3-DAP-Seq(GSE60143)/Homer	1e-2	-6.250e+00	0.0428	101.0	7.34%
45	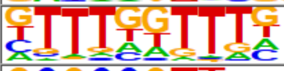	HuR(?)/HEK293-HuR-CLIP-Seq(GSE87887)/Homer	1e-2	-6.141e+00	0.0467	736.0	53.49%
46	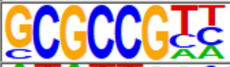	PUCHI(AP2EREBP)/colamp-PUCHI-DAP-Seq(GSE60143)/Homer	1e-2	-6.119e+00	0.0467	109.0	7.92%
47	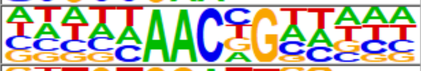	MYB73(MYB)/col-MYB73-DAP-Seq(GSE60143)/Homer	1e-2	-6.072e+00	0.0479	348.0	25.29%
48	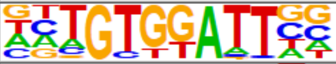	Foxh1(Forkhead)/hESC-FOXH1-ChIP-Seq(GSE29422)/Homer	1e-2	-5.974e+00	0.0517	121.0	8.79%
49	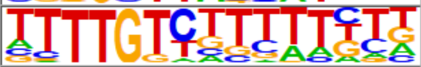	IDD5(C2H2)/colamp-IDD5-DAP-Seq(GSE60143)/Homer	1e-2	-5.945e+00	0.0521	154.0	11.19%
50	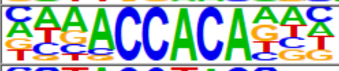	RUNX2(Runt)/PCa-RUNX2-ChIP-Seq(GSE33889)/Homer	1e-2	-5.926e+00	0.0521	115.0	8.36%
51	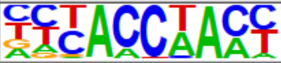	AT4G26030(C2H2)/col-AT4G26030-DAP-Seq(GSE60143)/Homer	1e-2	-5.787e+00	0.0587	182.0	13.23%
52	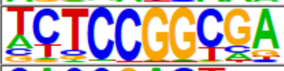	AT3G58630(Trihelix)/col-AT3G58630-DAP-Seq(GSE60143)/Homer	1e-2	-5.785e+00	0.0587	80.0	5.81%
53	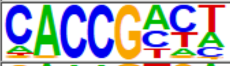	At1g75490(AP2EREBP)/colamp-At1g75490-DAP-Seq(GSE60143)/Homer	1e-2	-5.782e+00	0.0587	326.0	23.69%
54	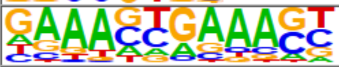	IRF2(IRF)/Erythroblas-IRF2-ChIP-Seq(GSE36985)/Homer	1e-2	-5.769e+00	0.0587	27.0	1.96%
55	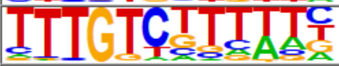	IDD4(C2H2)/col-IDD4-DAP-Seq(GSE60143)/Homer	1e-2	-5.744e+00	0.0587	181.0	13.15%
56	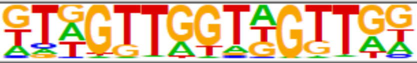	AT1G24250(Orphan)/col-AT1G24250-DAP-Seq(GSE60143)/Homer	1e-2	-5.711e+00	0.0587	78.0	5.67%
57	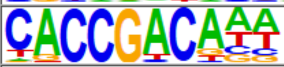	At4g31060(AP2EREBP)/colamp-At4g31060-DAP-Seq(GSE60143)/Homer	1e-2	-5.574e+00	0.0650	93.0	6.76%
58	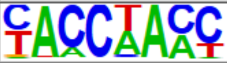	MYB55(MYB)/colamp-MYB55-DAP-Seq(GSE60143)/Homer	1e-2	-5.469e+00	0.0710	197.0	14.32%
59	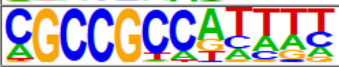	RAP212(AP2EREBP)/col-RAP212-DAP-Seq(GSE60143)/Homer	1e-2	-5.449e+00	0.0711	125.0	9.08%
60	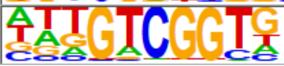	CEJ1(AP2EREBP)/col-CEJ1-DAP-Seq(GSE60143)/Homer	1e-2	-5.442e+00	0.0711	178.0	12.94%

61	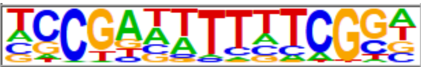	LBD2(LOBAS2)/colamp-LBD2-DAP-Seq(GSE60143)/Homer	1e-2	-5.401e+00	0.0722	95.0	6.90%
62	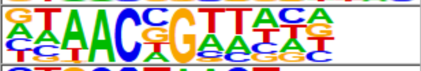	MYB81(MYB)/col-MYB81-DAP-Seq(GSE60143)/Homer	1e-2	-5.398e+00	0.0722	276.0	20.06%
63	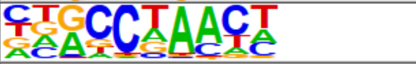	BOS1(MYB)/col-BOS1-DAP-Seq(GSE60143)/Homer	1e-2	-5.329e+00	0.0751	162.0	11.77%
64	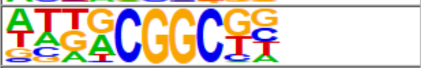	ERF115(AP2EREBP)/colamp-ERF115-DAP-Seq(GSE60143)/Homer	1e-2	-5.311e+00	0.0753	218.0	15.84%
65	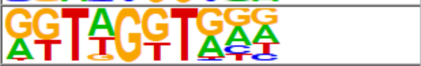	MYB93(MYB)/colamp-MYB93-DAP-Seq(GSE60143)/Homer	1e-2	-5.234e+00	0.0801	205.0	14.90%
66	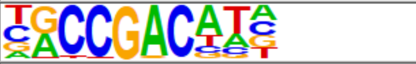	CBF1(AP2EREBP)/colamp-CBF1-DAP-Seq(GSE60143)/Homer	1e-2	-5.220e+00	0.0801	129.0	9.38%
67	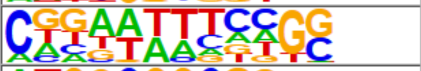	LBD18(LOBAS2)/colamp-LBD18-DAP-Seq(GSE60143)/Homer	1e-2	-5.113e+00	0.0877	425.0	30.89%
68	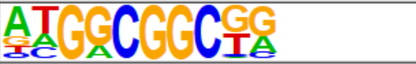	AT4G18450(AP2EREBP)/col-AT4G18450-DAP-Seq(GSE60143)/Homer	1e-2	-5.036e+00	0.0933	68.0	4.94%
69	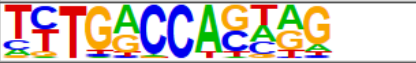	Bcl11a(Zf)/HSPC-BCL11A-ChIP-Seq(GSE104676)/Homer	1e-2	-4.980e+00	0.0972	67.0	4.87%
70	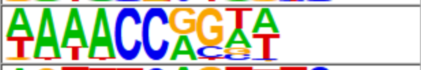	AT1G76870(Trihelix)/col-AT1G76870-DAP-Seq(GSE60143)/Homer	1e-2	-4.969e+00	0.0972	79.0	5.74%
71	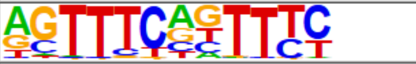	IRF3(IRF)/BMDM-Irf3-ChIP-Seq(GSE67343)/Homer	1e-2	-4.967e+00	0.0972	67.0	4.87%
72	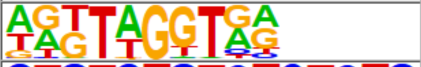	MYB49(MYB)/col-MYB49-DAP-Seq(GSE60143)/Homer	1e-2	-4.888e+00	0.1022	207.0	15.04%
73	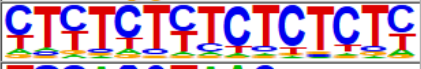	GAGA-repeat/SacCer-Promoters/Homer	1e-2	-4.844e+00	0.1053	669.0	48.62%
74	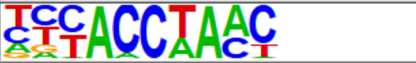	MYB67(MYB)/col-MYB67-DAP-Seq(GSE60143)/Homer	1e-2	-4.776e+00	0.1112	170.0	12.35%
75	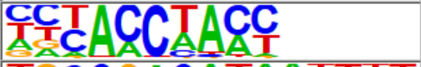	MYB61(MYB)/colamp-MYB61-DAP-Seq(GSE60143)/Homer	1e-2	-4.775e+00	0.1112	250.0	18.17%
76	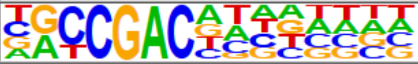	CBF4(AP2EREBP)/colamp-CBF4-DAP-Seq(GSE60143)/Homer	1e-2	-4.705e+00	0.1162	142.0	10.32%

Fig. S4. Overrepresented motifs at 2 h identified by positively enriched THSs.

Rank	Motif	Name	P-value	log P-pvalue	q-value (Benjamini)	# Target Sequences with Motif	% of Targets Sequences with Motif	# Background Sequences with Motif	% of Background Sequences with Motif
1	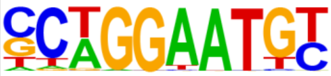	TEAD2(TEA)/Py2T-Tead2-ChIP-Seq(GSE55709)/Homer	1e-2	-5.035e+00	1.0000	15.0	7.94%	1901.6	3.83%

Fig. S5. Overrepresented motifs at 2 h identified by negatively enriched THSs.

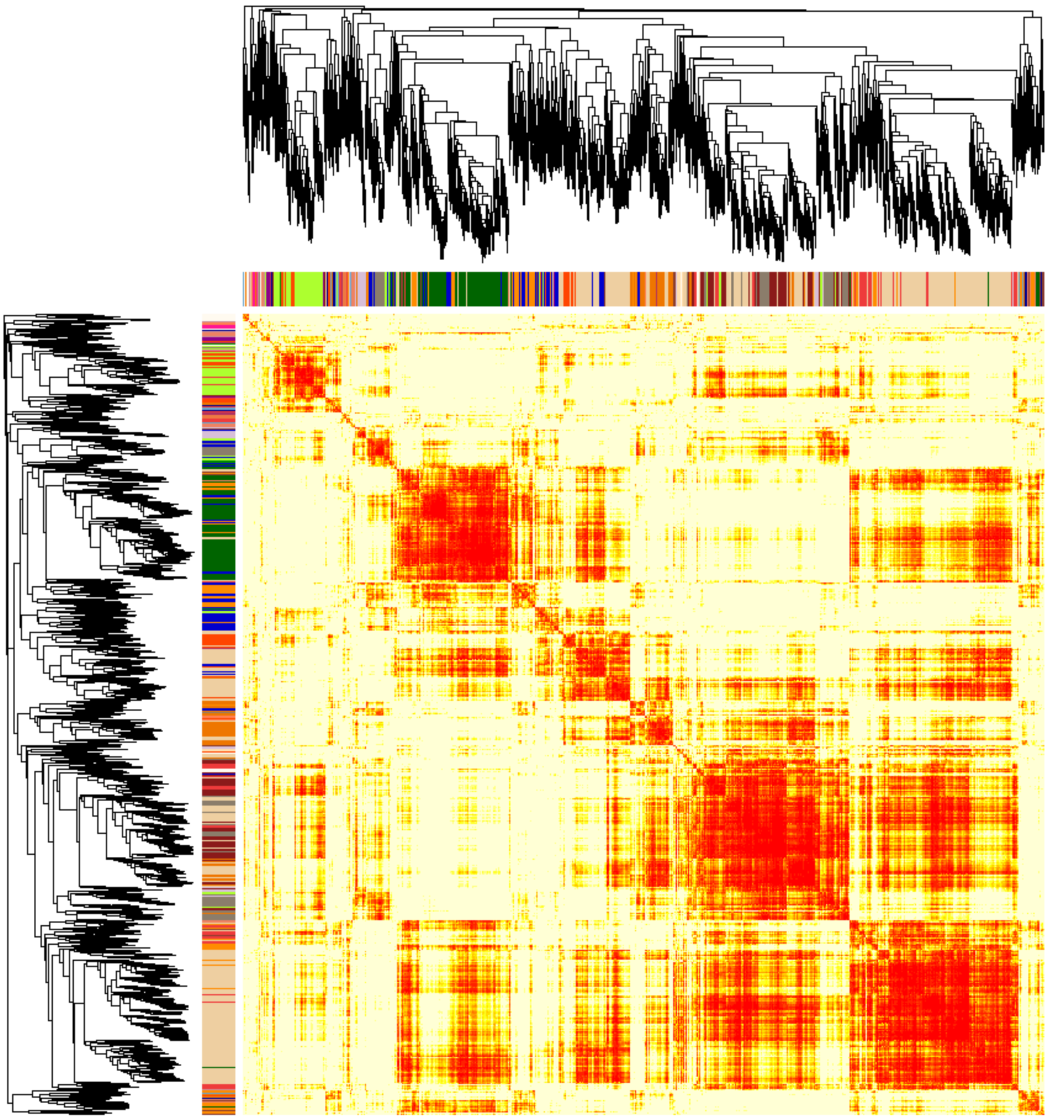

Fig. S6. The results of weighted gene co-expression network analysis (WGCNA) using RNA-seq data. The genes with similar expression patterns may have a high correlation relationship and can be clustered into the same modules.

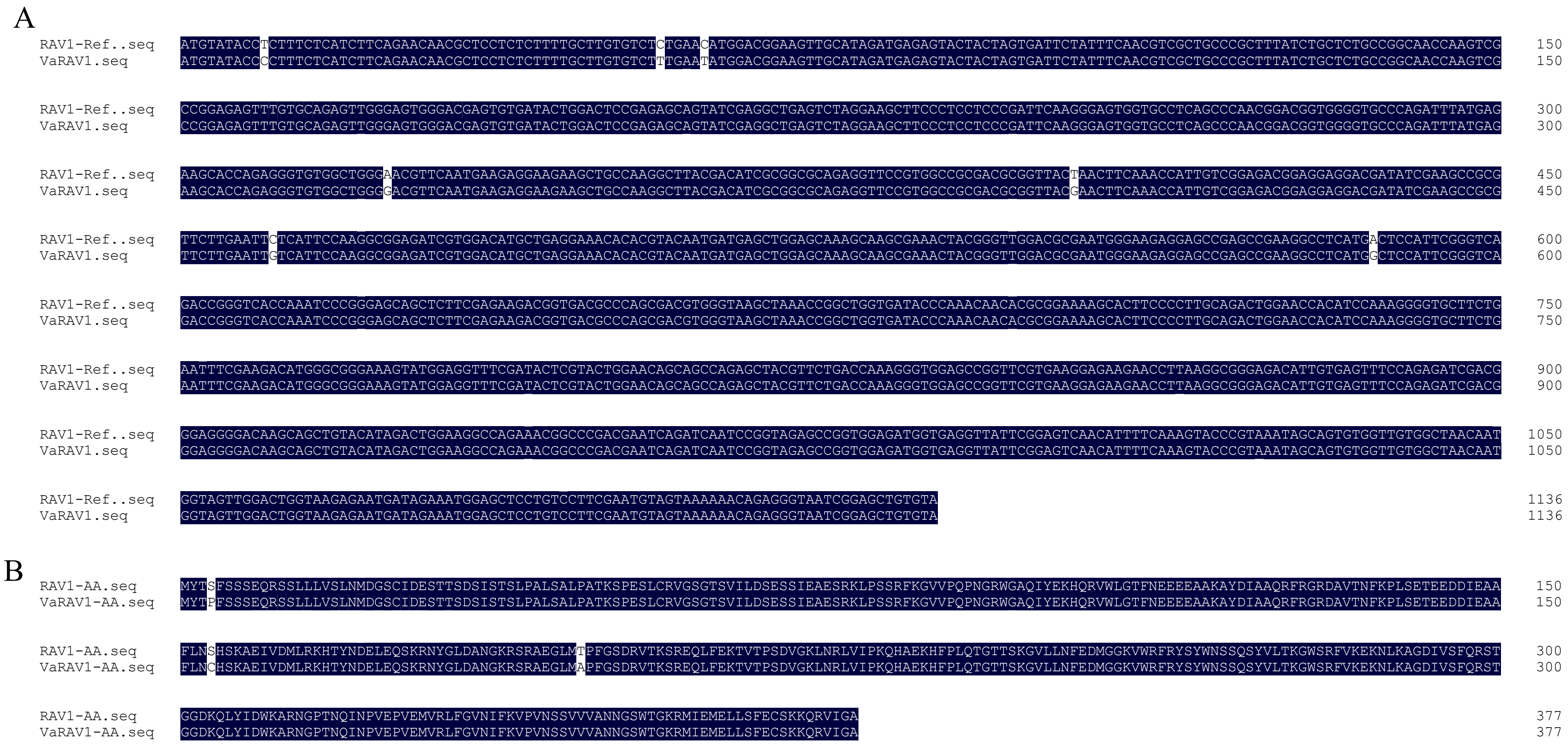

Fig. S7. Sequence of *VaRAV1*. (A) Alignment of the cloned *VaRAV1* with the reference (Ref) sequence. (B) Alignment of corresponding amino acids encoded by the sequences shown in (A). The identical sequences are highlighted in dark blue, while the changes are indicated in white.

A

```
ERF1A-Ref.seq    ATGTGTGATTACAGTAGTAATCCCTCTTCTGACTTCGCTCTCCTGGAGTCTGTTCGCCGCCACTTGTTCGACGACTCCGACTCTCGTCGCTTCGACGCTCCCCTGTATTGCCGTAGCAATAGTTTTAGTAGCTTGTTTGAAACTGGGGGT    150
VaERF1A.seq      ATGTGTGATTACAGTAGTAATCCCTCTTCTGACTTCGCTGTCCTGGAGTCTGTTCGCCGCCACTTGTTCGACGACTCCGACTCTCGTCGCTTCGACGCTCCCATGTATTGCCGTAGCAATAGTTTTAGTAGCTTGTTTGAAACTGGGGGT    150

ERF1A-Ref.seq    GAATTGCCTTTGAAGGAGGATGATTCCGACGACATGGTGATCTATGGCTTTCTCCGCGACGCTGCCATCGGAGGCTGGACACCCACGCTCGCTCCACTCTTTTCAGAGACTGCCTCCTACGGTTTCTCTGCCGCCCCCGCGGTGGCCGTG    300
VaERF1A.seq      GAATTGCCTTTGAAGGAGGATGATTCCGACGACATGGTGATCTATGGCTTTCTCCGCGACGCCGCCATCGGAGGCTGGACACCCACGCTCGCTCCACTCTTTTCAGAGACTGCCTCCTACGGTTTCTCTGCCGCCCCCGCGGTGGCCGTG    300

ERF1A-Ref.seq    AAATCGGAGCCGGAAGTTTTTCCGGCGGAGGTTATTGGAGTGCCCGAGAAGACGGTGGACCCACCGGCGAAATTACCTGCTCCGGCGGTGGTGCCGGCGAAGGGGAAGCATTACAGGGGCGTGCGGCAGAGGCCGTGGGGGAAGTTCGCT    450
VaERF1A.seq      AAATCGGAGCCGGAAGTTTTTCCGGCGGAGGTTGTTGGAGTGCCCGAGAAGACGGTGGAACCACCGGCGAAATTACCTGCTCCGGCGGTGGTGCCGGCGAAGGGGAAGCATTACAGGGGCGTGCGACAGAGGCCGTGGGGGAAGTTCGCT    450

ERF1A-Ref.seq    GCGGAGATTAGGGATCCGGCGAAGAACGGGGCTAGGGTTTGGCTGGGGACGTTCGAGACGGCAGAGGACGCCGCACTGGCTTATGACAGAGCCGCTTATCGGATGCGCGGCTCTCGCGCACTGCTCAATTTCCCACTCCGAGTTAACTCG    600
VaERF1A.seq      GCGGAGATTAGGGATCCGGCGAAGAACGGGGCTAGGGTTTGGCTGGGGACGTTCGAGACGGCAGAGGACGCCGCACTGGCCTATGACAGAGCCGCTTATCGGATGCGCGGCTCTCGCGCACTGCTCAATTTCCCACTCCGAGTTAACTCG    600

ERF1A-Ref.seq    GGGGAGCCCGATCCAGTCCGAGTGACCTCAAAACGATCCTCACCTGAGCCTTCCTCATCATCAACATCGTCATCATCATCAGATAATAGCTCACCCAAGCGTAGAAAGAAAGTGAGTAGCTTGGCTGCTCCCGCTGTGGCGCCGGCAACG    750
VaERF1A.seq      GGGGAGCCCGATCCAGTCCGAGTGACCTCAAAACGATCCTCACCTGAGCCTTCCTCTTCATCAACGTCGTCATCATCATCAGATAATAGCTCACCCAAGCGTAGAAAGAAAGTGAGTAGCTTGGCTGCTCCCGCTGTGGCGCCGGCAACG    750

ERF1A-Ref.seq    GCTCAAACTGGAATCGAAATAGGGAAGTCAATGGAGGGATCCCAGGCGGGATATGAGGTGGCACAGCTTACACATGGCCCACAGCTATTGGTTCGCTA    848
VaERF1A.seq      GCTCAAACTGGAATCGAAATAGGGAAGTCAATGGAGGGATCCCAGGCGGGATATGAGGTGGCACAGCTTACACATGGCCCACAGCTATTGGTTCGCTA    848
```

B

```
ERF1A-AA.seq      MCDYSSNPSSDFALLESVRRHLFDDSDSRRFDAPLYCRSNSFSSLFETGGELPLKEDDSDDMVIYGFLRDAAIGGWTPTLAPLFSETASYGFSAAPAVAVKSEPEVFPAEVIGVPEKTVDPPAKLPAPAVVPAKGKHYRGVRQRPWGKFA    150
VaERF1A-AA.seq    MCDYSSNPSSDFAVLESVRRHLFDDSDSRRFDAPMYCRSNSFSSLFETGGELPLKEDDSDDMVIYGFLRDAAIGGWTPTLAPLFSETASYGFSAAPAVAVKSEPEVFPAEVVGVPEKTVEPPAKLPAPAVVPAKGKHYRGVRQRPWGKFA    150

ERF1A-AA.seq      AEIRDPAKNGARVWLGTFETAEDAALAYDRAAYRMRGSRALLNFPLRVNSGEPDPVRVTSKRSSPEPSSSSTSSSSSDNSSPKRRKKVSSLAAPAVAPATAQTGIEIGKSMEGSQAGYEVAQLTHGPQLLV    281
VaERF1A-AA.seq    AEIRDPAKNGARVWLGTFETAEDAALAYDRAAYRMRGSRALLNFPLRVNSGEPDPVRVTSKRSSPEPSSSSTSSSSSDNSSPKRRKKVSSLAAPAVAPATAQTGIEIGKSMEGSQAGYEVAQLTHGPQLLV    281
```

Fig. S8. Sequence of *VaERF1A*. (A) Alignment of the cloned *VaERF1A* with the reference (Ref) sequence. (B) Alignment of corresponding amino acids encoded by the sequences shown in (A). The identical sequences are highlighted in dark blue, while the changes are indicated in white.

A

```
CRF2-Ref.seq   ATGTTGTCCCCAATCAAGTACAGCGAGCACCGCAAGCGTACTAAGCTGCTGACAACCTCGTCGGAGAAATTCACCGGCAGGAGTATGAATGCTGATGGGCCCAGAATTGTACGGATTTCCGTCACTGACGGTGACGCGACGGACTCTTCC   150
VaCRF2.seq     ATGTTGTCCCCAATCAAGTACAGTGAGCACCGCAAGCGTACTAAGCTGCTGACAACCTCGTCGGAGAAATTCACCGGCAGGAGTATGAATGCTGATGGGCCCAGAATTGTACGGATTTCCGTCACTGACGGTGACGCGACGGACTCTTCC   150

CRF2-Ref.seq   AGCGACGAAGAGGGTGAGCTTTTCGCGCGTCAGCGTGTGAAGAAGTTCGTTAACGAGATCACTATTCAGTCATGTTCAGGAGAGAACAGCGACGGCACCACTGTTTGGAGAAGCAGATCGGCGAGAAATGGCAGGAAGAAGTCCACCGGA   300
VaCRF2.seq     AGCGACGAAGAGGGTGAGCTTTTCGCGCGTCAGCGTGTGAAGAAGTTCGTTAACGAGATCACTATTCAGTCATGTTCAGGAGATAACAGCGACGGCACCACTGTTTGGAGAAGCAGATCGGCGAGAAATGGCCGGAAGAAGTCCACCGGA   300

CRF2-Ref.seq   AAAGGTGAAGCTCAGGCTGTCCGGCAGCTGGCGAAGCCGTCGACGGGTAAAAAGTTTCGCGGCGTTCGGCAACGTCCTTGGGGGAAATGGGCGGCTGAAATCAGAGATCCTCTGCGACGTGTACGGCTGTGGTTAGGTACCTATGATACG   450
VaCRF2.seq     AAAGGTGAAGCTCAGGCTGTCCGGCAGCTGGCGAAGCCGTCAACGGGTAAAAAGTTTCGCGGCGTTCGGCAACGTCCTTGGGGGAAATGGGCGGCTGAAATCAGAGATCCTCTGCGACGTGTACGGCTCTGGTTAGGTACCTATGATACG   450

CRF2-Ref.seq   GCGGAGGAGGCCGCCATGGTGTACGACAACGCAGCGATTCAGCTGCGTGGACCCGACGCGCTCACCAACTTCGCTTCACCCCCCGCGGTGACCAAGCCAAAAATAGAGCCGGTGACGGTGACGTGCTCCAGCTCCGGCGAAGAGTCTCAC   600
VaCRF2.seq     GCGGAGGAGGCCGCCATGGTGTACGACAACGCAGCGATTCAGCTGCGTGGACCCGACGCGCTCACCAACTTCGCTTCACCCCCCGCGGTGACAAAACCAAAAATAGAGCCGGTGACGGTGACGTGCTCCAGCTCCGGCGAAGAGTCTCAC   600

CRF2-Ref.seq   AACAATCTCTGCTCTCCGACCTCGGTTCTCCGATATATCAACGAAGAAGCCGAGTCA......CAAGGACCCATCCCCGAAACCCCGAAACAAACCCGAAGCACTGAACTACCCCGAATTATCGGAAACGACACAGGCGTATCGGATAAC   744
VaCRF2.seq     AACAATCTCTGCTCTCCGACCTCGGTTCTCCGATATATCAACGAAGAAGCCGAGTCGCAATCGCAAGGACCCATCCCCGAAACCCCGAAACAAACTCGAAGCACTGAACTACCCCGAATTATCGGAAACGACACAGGCGTATCGGATAAC   750

CRF2-Ref.seq   TTTTTGGATGTTTCGGAATTTGACACACTATTTGTTAACGATGCCTTCGATTTCCAAAGTTCAGTACCAGAGTTTTTCGGCGAGACGAATCTTCTAGACAGCATATTGAGAGAAGACTGCAGCGATATCTTCCTTAAATCGAGTGAAGAT   894
VaCRF2.seq     TTTTTGGATGTTTCGGAATTTGACACACTATTTGTTAACGATGCCTTCGATTTCCAAAGTTCAGTACCAGAGTTTTTCGGCGAGACGAATCTTCTAGACAGCATATTGAGAGAAGACTGCAGCGATATCTTCCTTAAATCGAGTGAAGAT   900

CRF2-Ref.seq   TTCGGATTCAGATTCCCGACGAGTCCCGTTGATGAGTATTTTCAAGACATCGGCGATTTATTCGTCTCCGACCCTCTCGTCGCGCTTTA   983
VaCRF2.seq     TTCGGATTCAGATTCCCGACGAGTCCCGTTGATGAGTATTTTCAAGACATCGGCGATTTATTCGTCTCCGACCCTCTCGTCGCGCTTTA   989
```

B

```
CRF2-AA.seq     MLSPIKYSEHRKRTKLLTTSSEKFTGRSMNADGPRIVRISVTDGDATDSSSDEEGELFARQRVKKFVNEITIQSCSGENSDGTTVWRSRSARNGRKKSTGKGEAQAVRQLAKPSTGKKFRGVRQRPWGKWAAEIRDPLRRVRLWLGTYDT   150
VaCRF2-AA.seq   MLSPIKYSEHRKRTKLLTTSSEKFTGRSMNADGPRIVRISVTDGDATDSSSDEEGELFARQRVKKFVNEITIQSCSGDNSDGTTVWRSRSARNGRKKSTGKGEAQAVRQLAKPSTGKKFRGVRQRPWGKWAAEIRDPLRRVRLWLGTYDT   150

CRF2-AA.seq     AEEAAMVYDNAAIQLRGPDALTNFASPPAVTKPKIEPVTVTCSSSGEESHNNLCSPTSVLRYINEEAESQG..PIPETPKQTRSTELPRIIGNDTGVSDNFLDVSEFDTLFVNDAFDFQSSVPEFFGETNLLDSILREDCSDIFLKSSED   298
VaCRF2-AA.seq   AEEAAMVYDNAAIQLRGPDALTNFASPPAVTKPKIEPVTVTCSSSGEESHNNLCSPTSVLRYINEEAESQSQGPIPETPKQTRSTELPRIIGNDTGVSDNFLDVSEFDTLFVNDAFDFQSSVPEFFGETNLLDSILREDCSDIFLKSSED   300

CRF2-AA.seq     FGFRFPTSPVDEYFQDIGDLFVSDPLVA   326
VaCRF2-AA.seq   FGFRFPTSPVDEYFQDIGDLFVSDPLVA   328
```

Fig. S9. Sequence of *VaCRF2*. (A) Alignment of the cloned *VaCRF2* with the reference (Ref) sequence. (B) Alignment of corresponding amino acids encoded by the sequences shown in (A). The identical sequences are highlighted in dark blue, while the changes are indicated in white.

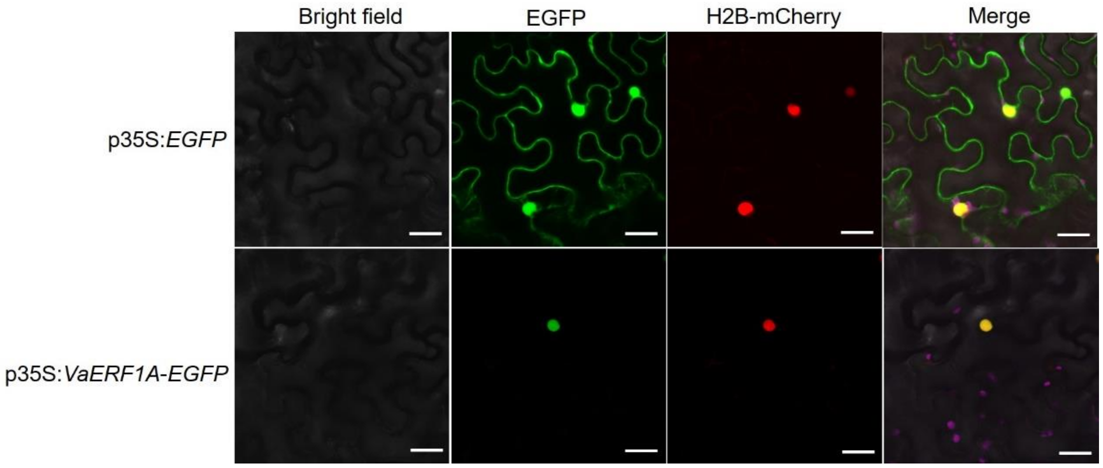

Fig. S10. Subcellular localization of the VaERF1A. The coding sequence of the VaERF1A fused to the N-terminal of EGFP gene was under the control of 35S promoter (p35S). The EGFP fluorescence generated by the p35S::VaRAV1-EGFP construct in epidermal cells of *Nicotiana benthamiana* leaves was detected using confocal laser scanning microscopy. The histone H2B-mCherry was used as an indicator of nucleus. Scale bars correspond to 30 μm.

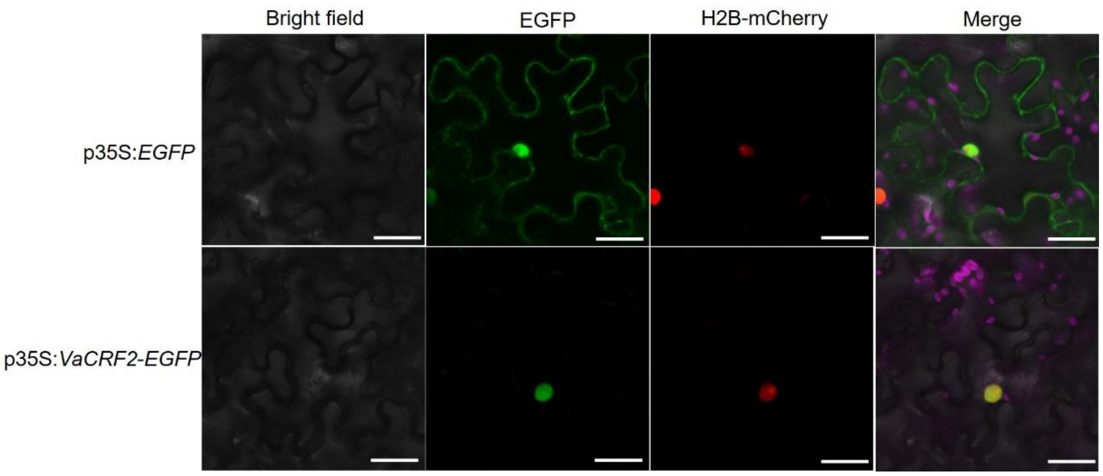

Fig. S11. Subcellular localization of the VaCRF2. The coding sequence of the VaCRF2 fused to the N-terminal of EGFP gene was under the control of 35S promoter (p35S). The EGFP fluorescence generated by the p35S::VaRAV1-EGFP construct in epidermal cells of *Nicotiana benthamiana* leaves was detected using confocal laser scanning microscopy. The histone H2B-mCherry was used as an indicator of nucleus. Scale bars correspond to 30 μm.

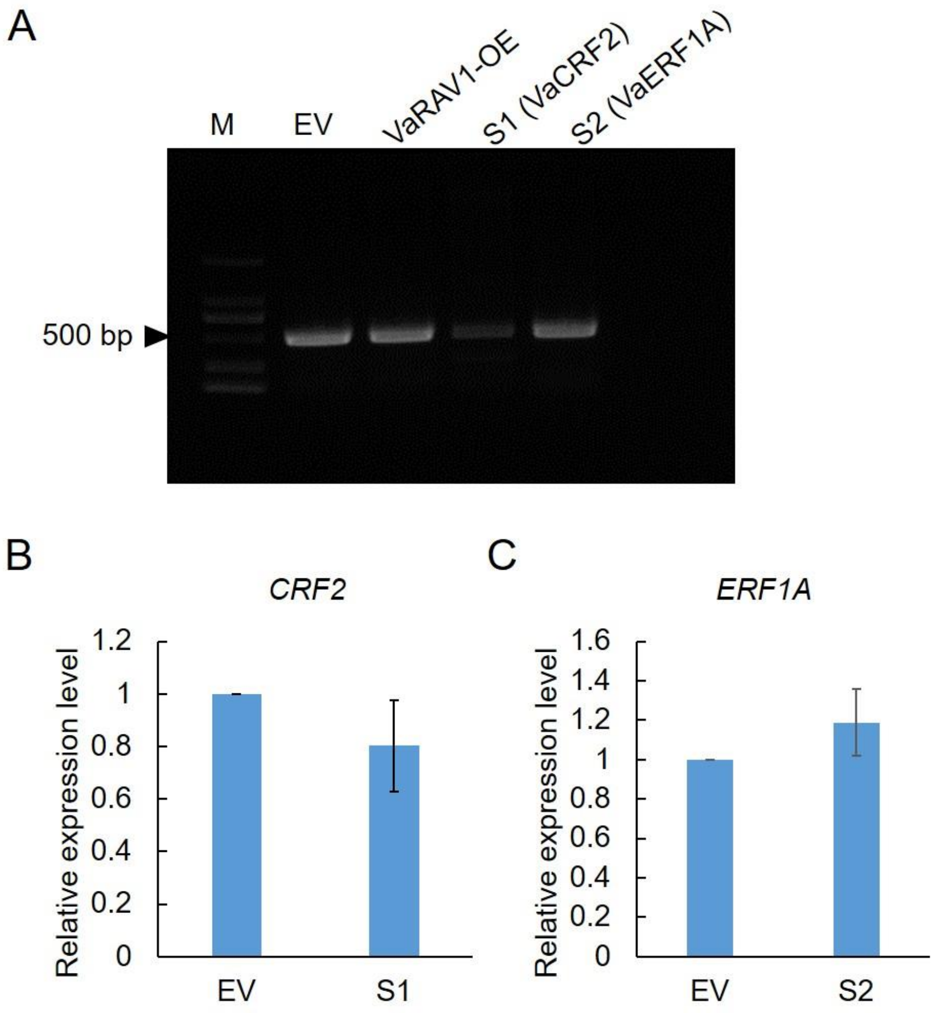

Fig. S12. Identification of VaERF1A- and VaCRF2-overexpressing cells. (A) PCR identification of exogenous T-DNAs using *EGFP*-specific primers in 41B cells. The grape cells transformed with empty vector (EV) and p35S::*VaRAV1-EGFP* construct (VaRAV1-OE) were used as the controls. The sample 1 (S1) and 2 (S2) are the cells that were transformed with p35S::*VaCRF2-EGFP* and p35S::*VaERF1A-EGFP* constructs, respectively. (B) Expression level of *CRF2* in S1 cells. (C) Expression level of *ERF1A* in S2 cells. The expression of *CRF2* or *ERF1A* in the EV cells was set as 1, and the transcript abundance of *CRF2* or *ERF1A* in the OE cells relative to the EV was determined by qPCR.

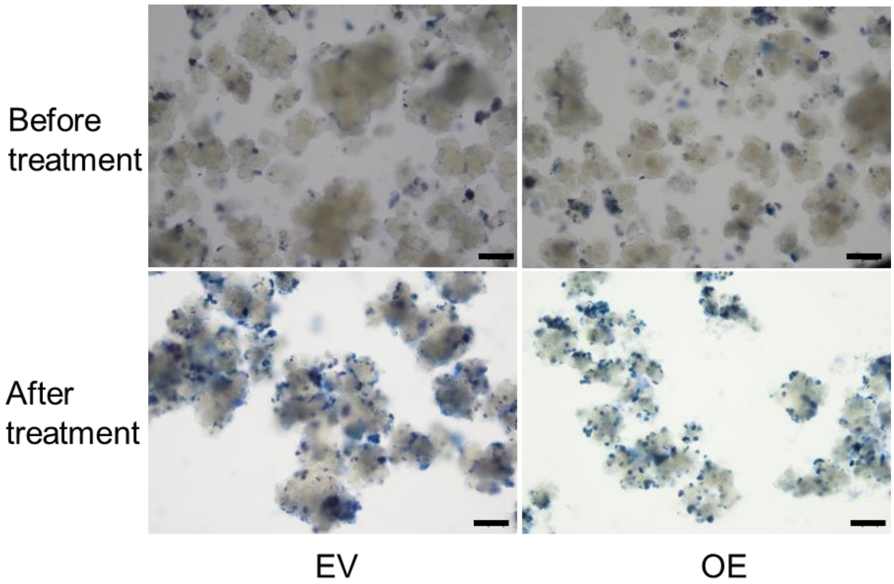

Fig. S13. Staining results of 41B cells. The *VaRAV1*-overexpressing (OE) cells and the cells transformed with empty vector (EV) were treated at freezing temperature (-4 ℃, 0.5 h), the cells before and after treatment were incubated with Trypan blue to stain the dead cells. Scare bars: 100 μm.

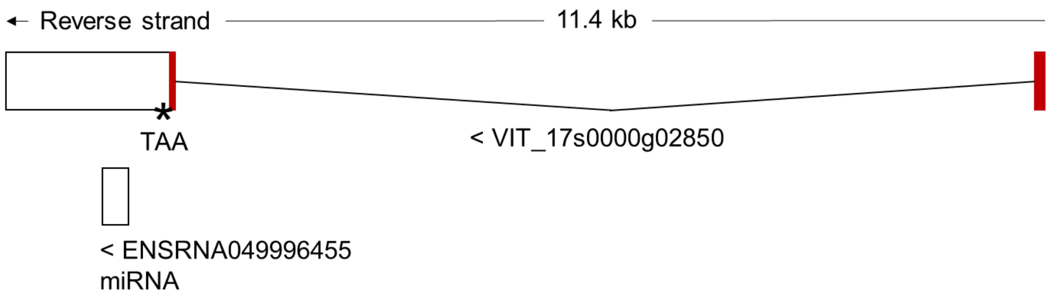

Fig. S14. Schematic diagram of the *VIT_17s0000g02850* gene. The exons are indicated in red boxes and the intron is indicated in black line. The miRNA gene (ENSRNA049996455) is located in the second exon of the *VIT_17s0000g02850* gene. The emergence of a premature stop codon (TAA, indicated in asterisk) results in the early termination of translation.

The promoter sequence of *VIT_218s0001g08770*:
ACGAGACACATAATAAATTATTATTTTTAATTTTATTATCAAAAGAACCTGTAAAATAATA
ACGTTTGAAGGACTTAAAAAACTTTTCTGAATAAATAAATGACAACAAATTCATATATA
AAATATAAAAATTAAAATTAAAAAAAAAAAAGAATTTTTTTTAAATGATTAAGTTGAGAT
AATACTTAATAAAAAAAATTACAATATTCAAAAAATAAAAAATAAAAAAATAAAATTTG
AAAAATGCAGCCTCTTTCCTTTTCAACAAGAGGCAAGAGGCGGCATCTGTCCACCTGG
CACCTCCATCCCGCAGGAATCTTGAACACACGTTTCAGATGGTTTGAACACACGTTTC
AGATGGTGTCCAAAGGCGAACCGGAGATATGAGGCTTTGATCTCATCCAAACCCATTTC
AATGGTGTAAGGCGAACTGGAGATATGAGTCTTTGATCTCATCCAAACCCATTTCAGCC
CACACGGAATCGTACATATCATATTTATTTCTGATTATTCACGCATGGACCTTGAAGGTA
GAGGGGTATGGCCCACACAATCAGCTCAAAGCTAACAAACTTTCCTTTTGAACTCATC
CAATCTCTCTTAACGCCTTGACCAAAAAAGGTTTGACAAAGTTTGAAAACCCCTATAAT
AATAAAATATTAAAAATGTTTAGATAAAAATGTTTTGGGCTGCAAAGTGGGCCAGGCGG
AGCCACGTACAAATGTCCAGAAACCAGAAAGCCTAATTTGAGGGAGGTAGGTTTTGGC
TCGAGTGGTATACCGTATACGTATGTTGCAGTATGTCTACCCTGTTGGAATCGAAGGTGC
GCATAACGCACCCTGTCGGGCTTGAATAACAAGTCGTCGCGC

The promoter sequence of *VIT_201s0026g00980*:
TGGTCCAGGCTGTAGCGTACAAGTGGGGGAGGAAGGAAAAGGTCGTCCACTGAAAGT
GTTGGAACTTCGGAGCACGTGATCACGTGGTGAGACGCCAGTGGATGGCAGCCGTCA
ACATCACTCAGAACACCAAACAGCTGGCTGCATCGGATGCACAGCTGGCGTCCCATTA
CTGGAGGAAAAGAATATTACTTGTTTCGAGCGCCGATTACCTCACCCGCTGCCCCACTC
GCCACCTCACCCCACCTCTTTTCCCTGCATGACACCTGGCACCCCCACTCTTTCCTACA
TTTAAAGTTAAAGAATGAAAAAGCCAAACCTATTACTGCAAGTCAAGTCAACTAATTTT
TCTTGATGCCCACCTCGCCGCGCTTTACTCCATCGATGTAAAGAATAGAAGAATGACTT
TGTGTGGAAGCCATGAAAAGTAGAAAATTTATTCTTTTTTATTTTAGAAAAAATATCAAC
AAAAAATCTTTTAATCCAATGCGAATCAAGACACGCAAACACGTTGAATCCATATTATA
CATTATAATTTTTTTATTTAAAAAAAAGTAATTAAAATATTAAAATTAAAGAAAAACCAA
ATATACAAAGTTTGAAGTTTGATAGGAAAAGAGAAAATCTTTTATAGTAAAATGTTTGG
AGCATGAAGGTTGGTATGACATTGATTTTGTAAAAGATGGTCCAAATATGAGTATGGAA
CATATTTATTATGGCAGCAAATACGTGTACAAGTAGAAGTGTGGGGGC

The promoter sequence of *VIT_218s0117g00340*:
CCTATACCTAGCCGTTCATGGCAATCAGATAAAAATTCCCCACCATGTTTAATCAAAATA
TATGGTGAAATAAATTGGAAATATTAAAAAAAAAAAAAAGATAAGAAATAATATTTGCA
ATATCATTTATGTTTTTTATTTTGGGTCCGATTATGATACATAAATATACTTTTTGATATTTA
TTAAAAGGACATTATCAATCAGATGGTATCATATATATATATATATTCTCAAGTCTCATTGT
CTGCATCTTAATCTCATTTATTTTTATTCTCGTTCAACGACCTAGATTACATCATACATATT
CGATCGATAATCAAAATATTTTGTTACCACCATGTTATCTTAGTATTTTTCTTTCACTTTTC
TCTTTTAACAAAAGATGTATTTGGACTCAGAGTTTTCATATAGGAATCAAAGTGTTATGT
TGGAAAAATAAAAGTGAACAAAATTACATCTTATGATGGAAGCATTAAAAGTTTTTCTA
CCTCCTTCACCATCTTGTTTTTAAATTTGAGTAAAATGCAAGAAAGAATAAATAATAAA
AATGTATTGATTTTTTTCCCCTTTATATCACTACTTGCTACTAATGTTCTAGAAGTAAAGG
GAATTGGCGGATCAGAGTGCTAATGATTTTAGTTTAAATCAAAAGTTAAAAGTTAGTCT

AAATCCAAGATGTTCACCTGAGACTGATTCTACTTGATTGATCATTCATTCATCACCTGAGACTGATGGTATAGATAAGTATTATTGTTTGAAGCATGTGAAATTGGAAAGCAATGGGATCCGAAGGGTTGAACCATGAGGGGAGAGTGTAAACCAGCGGTTACCAGCATTCATCCAATTGACATTCCTGGCAATGACCCCACGAGTGGGACACTGCTCCACTTACAGGTCCGCACCTGCAACCACTTGGACCCCACACTCCACCCACAAACAAGTAAACAACCACATCCCTCTCTTCTTCTCCCTTTAGTTTTATAGCCTCCCCCACAGGGTCATACCTGCTTCTCCCTTGCAACGCCAGTCAACCACACGCCACACAAAACCCAAACCCCACTCGCCCCTCCTTCCTCCTTCCCTCCCTGTACA

The promoter sequence of *VIT_207s0151g00800*:
GAACGATTTTACGCCGAAGCACTGCTGTGATCAGGACTACTTGACAATCACCTGAGTTGGGCCCACCATGTCTGACTGAACTTTGTGGACCCCACAAGCCCATACATTTTACATGGTCTGCAGGTGTCCACCGTTATTTCAGTGGGCTTGCAACCCAACCACTCCTGCCCTTCTCATTCGAACACCTAGGCTGCATAGTAGGTAAAAAAACAAGTAAATTAAGAATGTTTATTGGGCAGTTTTTTTTTTCCTTTTTAATATATATATTTTTTCATTTTGGGAAAATATTTCATATTGTATTTTAATTTATAATAAATTTAATATTTAAGGGTTTTCCAATAAAAACATTTTCAAAAAAATAAGGTGATTAAAATAATAATATATTTGATTTTTTTTTATAACATTAGTCATTAATGATGAGTAAGATATTGATTGTTATTAGGTGTACATGATATCATTTTTAATTATCTAGAATCAATTTTAAAATAGTAAAAATATTTTAAATGTATAATATAATTAATATTTTAAATAAATAAATAAATTTATTAAAAATAGATAAATATCTTAAATAAGTTAAAAATAATTAAATCATTTTTCCTATTTAATAAAAAAAAACAAACATCATCTTACAATTTTTTTGGGAGCACTTTAATATTTTAAAAAAAGGAAAAAAAAAAGGAGACCCATAGTTTTGGTAACTTAAAACTGAATGAATTTCATCAGATAGACTTTTCTAAATCTAAGGATTTTATGAGTTGGTGGTGAGCACTGTGAGTTTGAAAAAGGGAAACTTGTACGAGCAAGAAATGGGGATGAAGAAAGGTGTACGAGCAAGGGGATAACTCAGCCTTCAAGTGCGTGCCTTCAATTTAATTCTGCATTGAAGTGGGGAGAGAGAGGAGAGAGGACGGTGAAGAAGAGGGAGTCACGACAAATTCTCAGATAGGGAGACTGTG

Fig. S15. Promoter sequences for transient luciferase (Luc) assay. The predicted RAV1 motifs were highlighted in yellow. The nucleotides changes of the motifs in the promoter sequence of *VIT_201s0026g00980* is indicated in red.

Table S1. List of primers used in this study.

Primer set	Forward primer	Reverse primer	Experiment
Actin	CTTGCATCCCTCAGCACCTT	TCCTGTGGACAATGGATGGA	qPCR
GAPDH	TTCTCGTTGAGGGCTATTCCA	CCACAGACTTCATCGGTGACA	
CBF1	AACCCAACTGCACCATCTTC	CATCATCCCAGCTGAATCCT	
CBF2	ATGGACTTGGACCGTGAGTC	ATCGGGAAAATTGAGGGAAG	
CBF3	CCCTCATCCTCCTCTTCCTC	TCCCCAGCTGAAGATGACTT	
CBF4	GAACTCCGGGAAGTGGGTAT	TCCCTCCTCTCATCTTGCAT	
HOS1	CAAGGAAAAGCAGTTACGGG	CTCTATCATTCTATCCACCAAGCC	
RAV1	GCAGACTGGAACCACATCCA	ATGTACAGCTGCTTGTCCCC	
ERF1A	TCTATGGCTTTCTCCGCGAC	AACCGTAGGAGGCAGTCTCT	
CRF2	TTCAGGAGAGAACAGCGACG	GACAGCCTGAGCTTCACCTT	
ICE1a	GCAATGCCGGTCAGAATCAC	AACACGAAGACGACGAGTCC	
ICE1b	CCAGCAAGGGTGGAGGTAAG	AGCCTGCTGGATGTCCAATC	
ICE1c	TGTCTCAGAGTCAGAGCGGA	ACCCACAAACCATCGAGGAC	
VIT_201s0026g00980	CACAATGCAACACGGCTCAG	CCAAAGCCCAGCATTGTACC	
VIT_218s0001g08770	TTGCAGCACAACCTCTCTCC	CTCCGCGCTAAATCCAAAGC	
VIT_218s0117g00340	TGACTACCAGCGTTAGCACC	CACCCCACCCGAGAAATTCA	
VIT_207s0151g00800	TCAATGGAGAGGGCGCAAAT	GAAAGCGCGGATGGTTGAAG	
RAV1-pLB	TTCCACCCAACCCAGTTTCA	TTGCAACATTCACAACATGCC	Cloning of CDS
ERF1A-pLB	TCGAACACACCAAACAAGAAGC	TGGGGAAATCTGTCTTTGCGA	
CRF2-pLB	GCTTTCCGTACCGTTTGTGC	GGAATTGGAAAAGCGGGCAT	
VIT_201s0026g00980-pLB	ATCCAGAGTGCTAACCGCTTT	CGGCTTCCATTGCCTAGTGT	Cloning of promoter
VIT_218s0001g08770-pLB	TGAGGTTCACATTTGTGCCAAC	GCGCGACGACTTGTTATTCA	
VIT_218s0117g00340-pLB	TGCAACTCTTCAAAGGCCAAC	CATCAGACCCATGTCCCAGC	
VIT_207s0151g00800-pLB	ACGATTTTACGCCGAAGCAC	AACCCACAACCAGTGTGGAC	
RAV1-2300	ACGGGGGACGAGCTCGGTACCTTCCACCCAACCCAGTTTCA	TCTAGAGGATCCCCGGGTACCTTGCAACATTCACAACATGCC	Construction of vector for overexpression/subcellular localization
ERF1A-2300	ACGGGGGACGAGCTCATGTGTGATTACAGTAGTAA	TCTAGAGGATCCCCGGCGAACCAATAGCTGTGGGC	
CRF2-2300	ACGGGGGACGAGCTCATGTTGTCCCCAATCAAGTA	TCTAGAGGATCCCCGAAGCGCGACGAGAGGGTCGG	
VIT_201s0026g00980-Luc	CGAGCTCGGTACCCGGGGATCCTGGTCCAGGCTGTAGCGTAC	ACTAGTGGGCCCAGGCCTAAGCTTGCCCCCACACTTCTACTTGT	Transient luciferase activity assay
VIT_218s0001g08770-Luc	CGAGCTCGGTACCCGGGGATCCACGAGACACATAATAAATTA	ACTAGTGGGCCCAGGCCTAAGCTTGCGCGACGACTTGTTATTCA	
VIT_218s0117g00340-Luc	CGAGCTCGGTACCCGGGGATCCCCTATACCTAGCCGTTCATG	ACTAGTGGGCCCAGGCCTAAGCTTTGTACAGGGAGGGAAGGAG	
VIT_207s0151g00800-Luc	CGAGCTCGGTACCCGGGGATCCGAACGATTTTACGCCGAAGC	ACTAGTGGGCCCAGGCCTAAGCTTGAGAAGTGGCGTGTGCATG	
RAV1-AD	TGCCAGATTATGCCTCTCCCTTCCACCCAACCCAGTTTCA	GCGAAGAAGTCCAAAGCTTCTTGCAACATTCACAACATGCC	Y1H assay
VIT_201s0026g00980-pHIS	AATTCCGCTACCCCACTCCACCT	GAGTGGGGGTGCCAGGTGGAGTG	

	GGCACCCCCACTCAGCT	GGGTAGCGG	
VIT_218s0001g08770-pHIS	AATTGCGGCATCTGTCCACCTGGCACCTCCATCCCGAGCT	CGGGATGGAGGTGCCAGGTGGACAGATGCCGC	
VIT_218s0117g00340-pHIS	AATTGTTCACCTGAGACTGATTCATCACCTGAGACTGAGCT	CAGTCTCAGGTGATGAATCAGTCTCAGGTGAAC	
VIT_207s0151g00800-pHIS	AATTGGACTACTTGACAATCACCTGAGTTGGGCCCACCAGCT	GGTGGGCCCAACTCAGGTGATTGTCAAGTAGTCC	
VIT_200s0228g00005-pHIS	AATTTCATCCGCTTACCACCTGGGCTAAGTTGCCCAGCT	GGGCAACTTAGCCCAGGTGGTAAGCGGATGA	
VIT_17s0000g02850-pHIS	AATTCTCCTTCCAGTAAGCAACATAAACTGATGCTTCCAGCT	GGAAGCATCAGTTTATGTTGCTTACTGGAAGGAG	
VIT_211s0016g04540-pHIS	AATTCTGTTTTCTCAGCAACAGAAAGAGAAAGTAGAGCT	CTACTTTCTCTTTCTGTTGCTGAGAAAACAG	
VIT_219s0090g01750-pHIS	AATTTAATGCAGAGGCAGCAACATAAGCTCACTTCAGCT	GAAGTGAGCTTATGTTGCTGCCTCTGCATTA	
EGFP-PCR	ACGTAAACGGCCACAAGTTC	GGTGTTCTGCTGGTAGTGGT	Identification of T-DNA

Table S2. Overview of the ATAC-seq data.

**Time**	**0 h**			**2 h**		
Sample	Rep.1	Rep.2	Rep.3	Rep.1	Rep.2	Rep.3
Total reads (No.)	69,726,666	71,931,332	60,544,807	55,684,534	66,603,189	46,858,097
Unique mapped reads (No.)	46,995,411	50,567,328	43,708,503	39,467,782	46,842,677	32,426,200
Unique mapped rate (%)	67.4	70.3	72.2	70.9	70.3	69.2
No. of Peaks/THSs	18,770	19,537	13,019	13,350	15,694	13,942

Table S3. Predicted target genes for RAV1.

Gene ID	Motif No.	Motif sequence	Log2FC	Annotation
VIT_217s0000g02850	1	AAGCAACATAAA	-1.24	NA
VIT_201s0026g00980	1	TCCACCTGGCAC	1.64	Galacturonosyltransferase-like 9
VIT_218s0001g08770	1	TCCACCTGGCAC	1.20	PHLOEM PROTEIN 2-LIKE A10
VIT_218s0117g00340	2	ATCACCTGAGAC; TTCACCTGAGAC	2.04	Transcription factor TCP8
VIT_207s0151g00800	1	ATCACCTGAGTT	1.19	MARD1-like
VIT_200s0228g00005	1	ACCACCTGGGCT	-2.32	NA
VIT_211s0016g04540	1	CAGCAACAGAAA	-1.33	NA
VIT_219s0090g01750	1	CAGCAACATAAG	-1.05	NA
